# Homologues of the Chlamydia trachomatis and Chlamydia muridarum Inclusion Membrane Protein IncS Are Interchangeable for Early Development but Not for Inclusion Stability in the Late Developmental Cycle

**DOI:** 10.1128/msphere.00003-23

**Published:** 2023-02-28

**Authors:** María Eugenia Cortina, Isabelle Derré

**Affiliations:** a Department of Microbiology, Immunology, and Cancer Biology, University of Virginia School of Medicine, Charlottesville, Virginia, USA; University of Kentucky

**Keywords:** *Chlamydia* developmental cycle, *Chlamydia* genome editing, *Chlamydia muridarum*, *Chlamydia trachomatis*, ER-inclusion membrane contact sites, effector proteins, FRAEM/FLAEM, IncS/CTL0402/TC0424, inclusion membrane proteins, STIM1, species specificity, temporal mutant complementation

## Abstract

Chlamydia trachomatis is an obligate intracellular bacterium, which undergoes a biphasic developmental cycle inside a vacuole termed the inclusion. Chlamydia-specific effector proteins embedded into the inclusion membrane, the Inc proteins, facilitate inclusion interaction with cellular organelles. A subset of Inc proteins engages with specific host factors at the endoplasmic reticulum (ER)-inclusion membrane contact site (MCS), which is a discrete point of contact between the inclusion membrane and the endoplasmic reticulum (ER). Here, we report that the C. trachomatis Inc protein CTL0402/IncS_Ct_ is a novel component of the ER-inclusion MCS that specifically interacts with and recruits STIM1, a previously identified host component of the ER-inclusion MCS with an unassigned interacting partner at the inclusion membrane. In comparison, the Chlamydia muridarum IncS homologue (TC0424/IncS_Cm_) does not interact with or recruit STIM1 to the inclusion, indicating species specificity. To further investigate IncS function and overcome the recently reported early developmental defect of the *incS* mutant, we achieved temporal complementation by expressing IncS exclusively during the early stages of the developmental cycle. Additionally, we used allelic exchange to replace the *incS_Ct_* open reading frame with *incS_Cm_* in the C. trachomatis chromosome. Inclusions harboring either of these strains progressed through the developmental cycle but were STIM1 negative and displayed increased inclusion lysis 48 h postinfection. Expression of *incS_Ct_* in *trans* complemented these phenotypes. Altogether, our results indicate that IncS is necessary and sufficient to recruit STIM1 to C. trachomatis inclusion and that IncS plays an early developmental role conserved in C. trachomatis and C. muridarum and a late role in inclusion stability specific to C. trachomatis.

**IMPORTANCE** Obligate intracellular pathogens strictly rely on the host for replication. Specialized pathogen-encoded effector proteins play a central role in sophisticated mechanisms of host cell manipulation. In Chlamydia, a subset of these effector proteins, the inclusion membrane proteins, are embedded in the membrane of the vacuole in which the bacteria replicate. Chlamydia encodes 50 to 100 putative Inc proteins. Many are conserved among species, including the human and mouse pathogens Chlamydia trachomatis and Chlamydia muridarum, respectively. However, whether the function(s) of Inc proteins is indeed conserved among species is poorly understood. Here, we characterized the function of the Inc protein IncS conserved in C. trachomatis and C. muridarum. Our work reveals that a single effector protein can play multiple functions at various stages of the developmental cycle. However, these functions are not necessarily conserved across species, suggesting a complex evolutionary path among Chlamydia species.

## INTRODUCTION

Chlamydia trachomatis is the leading cause of sexually transmitted infections of bacterial origin and therefore a human pathogen of tremendous public health concern (1). Infections are often asymptomatic, and if they are left untreated, the bacteria ascend to the upper genital tract, causing tissue inflammation that can lead to pelvic inflammatory disease, ectopic pregnancy, and infertility (2). Vaccines are not available (3, 4). Reinfection rates are high and contribute to the aggravation of the long-term consequences associated with infection due to the short-lived immunity acquired from infection (5).

C. trachomatis is an obligate intracellular bacterium that replicates in the host cell cytosol of genital epithelial cells within a membrane-bound compartment called the inclusion (6). Within the lumen of the inclusion, the bacteria undergo a characteristic biphasic developmental cycle, alternating between infectious elementary bodies (EBs) and replicating reticulate bodies (RBs) (6, 7). The developmental cycle begins with the entry of EBs into epithelial cells of the targeted tissue. The nascent inclusions traffic along microtubules to the microtubule organizing center (8). Concomitantly and within 8 h postentry, EBs differentiate into the initial RBs which will go on to replicate to high numbers. Toward midcycle, the inclusion occupies most of the cytosol and the RBs asynchronously transition back to EBs (7). Upon completion of the cycle, inclusions mostly contain EBs that can be released from the host cell either by lysis, whereby the inclusion and host cell burst, or by extrusion, whereby the inclusion, surrounded by the host plasma membrane, is extruded from the host cell (9, 10). In cultured cells, the C. trachomatis developmental cycle lasts 2 to 3 days.

To establish its replicative niche, Chlamydia remodels the membrane of the inclusion. This process involves the Chlamydia inclusion membrane proteins (Inc proteins), which are type III effector proteins that are translocated to and embedded within the inclusion membrane to mediate interactions between the inclusion and the host (11–15). Chlamydia encodes ~50 putative Inc proteins, many of which are conserved among species. A list of Inc and host protein interactions was described and partially validated (11, 16). However, the function of most Inc proteins remains elusive.

The inclusion membrane interacts with the host endoplasmic reticulum (ER) through points of membrane contacts without fusion which are referred to as ER-inclusion membrane contact sites (MCS) based on their similarities to MCS between cellular organelles (17–19). Characterization of the protein composition of ER-inclusion MCS led to the identification of two complexes, each composed of a specific Inc protein and host factors. One complex is composed of the Inc protein IncD, the ceramide transfer protein CERT (20), and the ER-resident protein VAP (21) and is proposed to participate in sphingomyelin acquisition by mediating the nonvesicular transfer of ceramide from the ER to the inclusion (17, 22, 23). Another complex consists of the Inc protein IncV and the ER resident host protein, VAP and constitutes a structural component that tethers the ER to the inclusion membrane (24). The assembly of the IncV-VAP complex is subjected to regulation via posttranslational phosphorylation by the host kinase CK2 (25).

The host protein stromal interaction molecule 1 (STIM1) is also enriched at ER-inclusion MCS (26, 27). STIM1 is a single-pass ER membrane protein important for maintaining calcium (Ca^2+^) homeostasis via store operated calcium entry (SOCE). Upon depletion of the ER Ca^2+^ store, STIM1 oligomerizes and relocalizes to ER-plasma membrane (PM) MCS where direct interaction with and activation of the Orai1 Ca^2+^ channel leads to extracellular Ca^2+^ influx and replenishment of the ER store (28). Orai1 does not localize to ER-inclusion MCS (26), suggesting that STIM1 function at the inclusion is Orai1 independent and that another factor is responsible for STIM1 recruitment to the ER-inclusion MCS.

Here, we show that the C. trachomatis inclusion membrane protein IncS (CTL0402) specifically recruits STIM1 to ER-inclusion MCS via direct interaction with a cytosolic domain of STIM1. Further characterization of the C. trachomatis and C. muridarum IncS homologues revealed that IncS performs an early developmental role conserved in C. trachomatis and C. muridarum and a late role in inclusion stability specific to C. trachomatis.

## RESULTS

### IncS_Ct_ specifically interacts with and recruits STIM1 to the Chlamydia trachomatis inclusion membrane.

**FIG 1:**
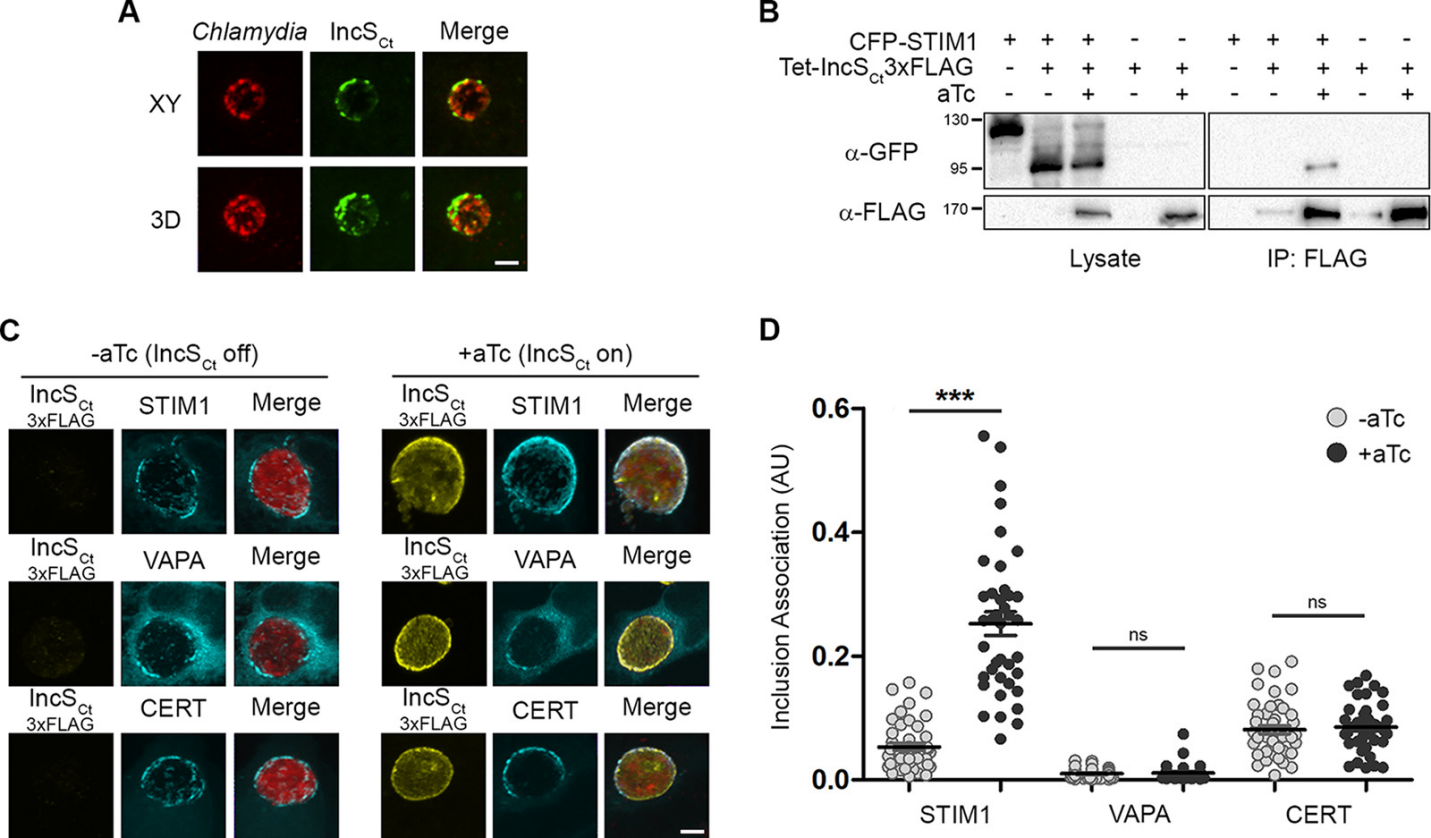
IncS_Ct_ interacts with and specifically recruits STIM1 to the inclusion membrane. (A) Confocal micrographs of HeLa cells infected for 24 h with C. trachomatis expressing mCherry constitutively (red, left panels) and stained for endogenous IncS_Ct_ (green, middle panels). Right panels, merge image; upper panels, single plane; lower panels, 3-dimensional reconstruction. Scale bar, 5 μm. (B) Coimmunoprecipitation (IP) of IncS_Ct_-3×FLAG from lysates of HEK293 cells expressing CFP-STIM1 and infected for 24 h with C. trachomatis expressing IncS_Ct_-3×FLAG under the control of an aTc-inducible promoter. Numbers at left are molecular masses in kilodaltons. (C) Three-dimensional confocal micrographs of HeLa cells expressing CFP-STIM1, CFP-VAPA, or CFP-CERT (blue) infected for 24 h with a C. trachomatis strain expressing mCherry constitutively (red) and IncS_Ct_-3×FLAG (yellow) under the control of an aTc-inducible promoter, in the absence (−aTc) or the presence (+aTc) of aTc. Scale bar, 5 μm. (D) Quantification of CFP-STIM1, CFP-VAPA, and CFP-CERT association with the inclusion when IncS_Ct_ expression is induced (+aTc, black dots) or not (−aTc, gray dots) in arbitrary units (AU), as in panel C. Each dot represents an inclusion. *n* = 40 inclusions. Unpaired *t* test, ***, *P* < 0.0001; ns, not significant.

To identify the STIM1-interacting partner at the inclusion membrane, we performed a pilot experiment with coimmunoprecipitation (co-IP) followed by mass spectrometry. Lysates from HEK293 cells expressing FLAG-tagged STIM1 and infected, or not, with wild-type C. trachomatis for 24 h were immunoprecipitated (IP) using anti-FLAG antibodies. Analysis of the samples by mass spectrometry revealed the presence of the C. trachomatis inclusion membrane protein CTL0402 in the coimmunoprecipitate of the infected samples. We have recently renamed CTL0402 IncS_Ct_ (29); this nomenclature will be used here. To confirm the inclusion localization of endogenous IncS_Ct_, HeLa cells infected with wild-type C. trachomatis for 24 h were fixed and stained with anti-IncS antibodies. Confocal microscopy revealed the presence of IncS_Ct_-positive patches, reminiscent of MCS, associated with the inclusion (Fig. 1A). To validate the IncS_Ct_-STIM1 interaction, we first generated a wild-type C. trachomatis strain expressing mCherry constitutively and IncS_Ct_ fused to a 3×FLAG tag under the control of an anhydrotetracycline (aTc)-inducible promoter and confirmed the aTc-dependent inclusion localization of IncS_Ct_ (see Fig. S1A and B in the supplemental material). We next performed co-IP experiments. HEK293 cells expressing cyan fluorescent protein (CFP)-STIM1 were infected for 24 h with C. trachomatis encoding the inducible IncS_Ct_-3×FLAG, in the presence or absence of aTc. Untransfected and/or uninfected cells served as controls. Lysates were immunoprecipitated using anti-FLAG-conjugated beads. Total lysates and IP samples were analyzed by Western blotting (WB) using anti-green fluorescent protein (GFP) antibodies, to detect CFP-STIM1, and anti-FLAG antibodies, to detect IncS_Ct_ (Fig. 1B). CFP-STIM1 was detected only in transfected and infected samples upon induction of IncS_Ct_ expression (Fig. 1B, lane 3, IP-FLAG blot). These results show that IncS_Ct_ interacts with STIM1.

10.1128/msphere.00003-23.1 FIG S1 aTc-inducible expression and inclusion localization of IncS_Ct_ in wild-type Chlamydia trachomatis expressing mCherry or GFP constitutively. (A and C) Cartoon depicting some of the elements included in the plasmid carried by each strain. (B and D) Single-plane confocal micrographs of HeLa cells infected for 24 h with a C. trachomatis strain expressing mCherry (C, red) or GFP (D, green) constitutively and IncS_Ct_-3×FLAG (C, yellow; B, red) under the control of an aTc-inducible promoter, in the absence (−aTc) or the presence (+aTc) of aTc. The merge is shown on the right. Scale bar, 5 μm. Download FIG S1, TIF file, 13.8 MB. Copyright © 2023 Cortina and Derré. 2023 Cortina and Derré. https://creativecommons.org/licenses/by/4.0/ This content is distributed under the terms of the Creative Commons Attribution 4.0 International license.

To address whether IncS_Ct_ was sufficient to recruit STIM1 to the inclusion membrane, HeLa cells expressing CFP-STIM1 were infected with the C. trachomatis IncS_Ct_-3×FLAG-inducible strain, in the absence or presence of aTc. The samples were fixed 24 h postinfection (p.i.), stained with anti-FLAG antibodies, and analyzed by confocal microscopy (Fig. 1C, upper panels). In the absence of aTc, IncS_Ct_ was not detected at the inclusion membrane and there were small STIM1 patches on the inclusion, as previously reported (26) (Fig. 1C, upper left panels, −aTc). In the presence of aTc, IncS_Ct_ localized to the inclusion membrane, leading to an apparent increase in STIM1 recruitment to the inclusion and to the colocalization of IncS_Ct_ with STIM1 (Fig. 1C, upper right panels, +aTc). Quantification of STIM1 association with the inclusion in the absence or presence of aTc confirmed that STIM1 recruitment to the inclusion was significantly increased upon IncS_Ct_ expression (Fig. 1D, STIM1). These results indicate that IncS_Ct_ expression leads to STIM1 recruitment to ER-inclusion MCS.

Since we previously reported that STIM1 colocalized with VAP and CERT at ER-inclusion MCS (26), we next investigated whether IncS_Ct_ also affected VAP and CERT inclusion association. HeLa cells expressing CFP-VAPA or CFP-CERT were infected with the C. trachomatis IncS_Ct_-3×FLAG-inducible strain. The samples were fixed 24 h p.i., stained with anti-FLAG antibodies, and analyzed by confocal microscopy (Fig. 1C, middle and lower panels). In the absence of aTc, small VAPA and CERT patches were observed on the inclusion (Fig. 1C, middle and lower left panels, respectively), which is in line with previous reports (17, 24). Addition of aTc did not increase the apparent recruitment of VAPA or CERT to the inclusion (Fig. 1C, middle and lower right panels), which was confirmed by quantification of VAPA and CERT association with the inclusion (Fig. 1D, VAPA and CERT).

Altogether, these results indicate that IncS_Ct_ is a novel component of ER-inclusion MCS that specifically recruits STIM1 to the inclusion membrane.

### A STIM1 region between aa 234 and 535 is necessary and sufficient for the IncS_Ct_-dependent recruitment of STIM1 to the inclusion membrane.

**FIG 2:**
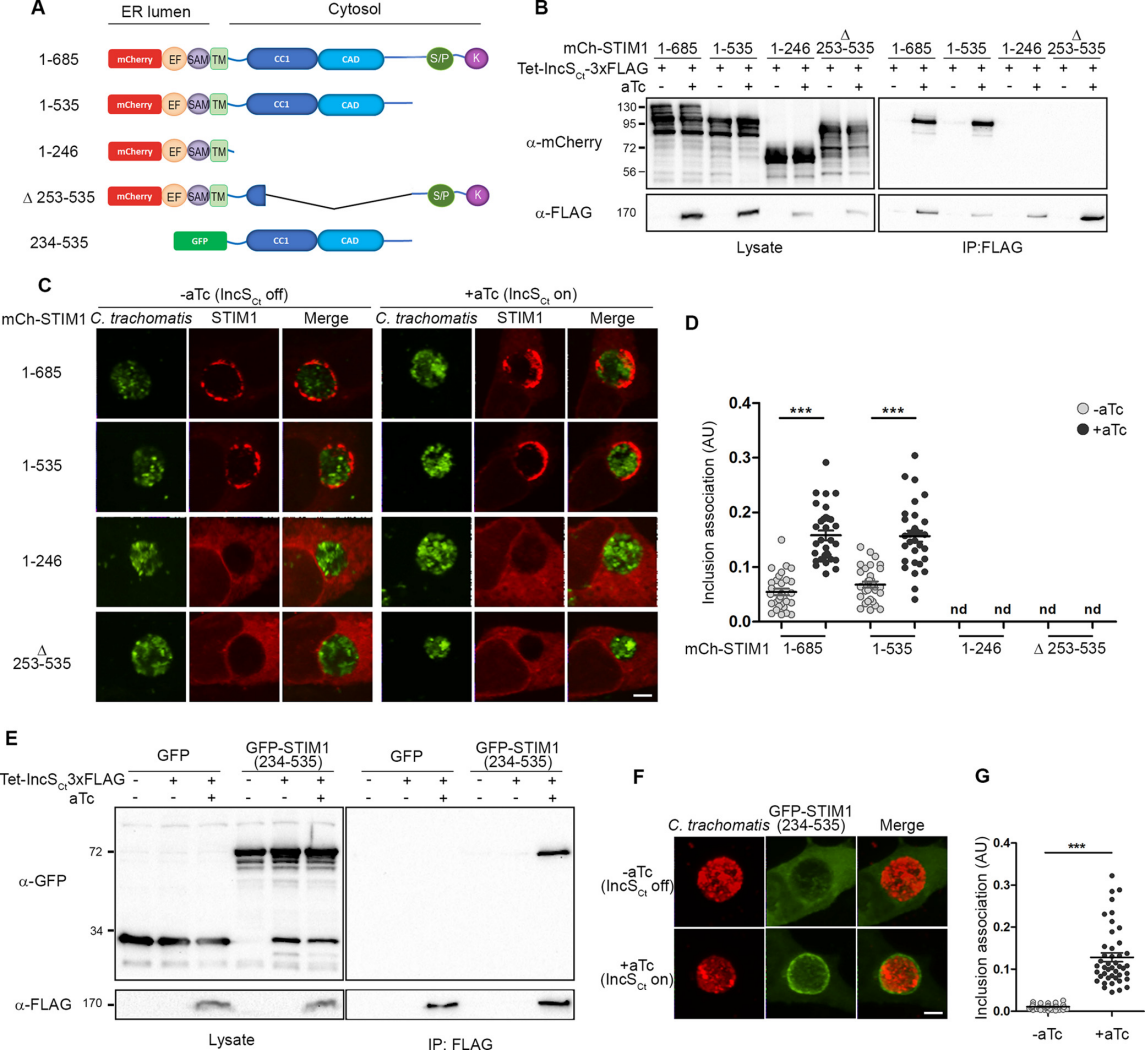
A STIM1 region between amino acids 234 and 535 is necessary and sufficient for the IncS_Ct_-dependent recruitment of STIM1 to the inclusion membrane. (A) Schematic representation of the STIM1 constructs employed in this study, indicating the major domains of the STIM1 protein, their respective cellular localization (ER lumen or cytosol), and the amino acid residue in the truncated protein. EF, EF-hand; SAM, sterile alpha motif; TM, transmembrane domain; CC1, coiled-coil 1; CAD, CRAC activation domain; S/P, serine-proline-rich region; K, lysine-rich region. (B) Coimmunoprecipitation (IP) of IncS_Ct_-3×FLAG from lysates of HEK293 cells expressing the indicated mCherry-STIM1 constructs and infected for 24 h with C. trachomatis expressing IncS_Ct_-3×FLAG under the control of an aTc-inducible promoter in the absence (−aTc) or the presence (+aTc) of aTc. Numbers at left are molecular masses in kilodaltons. (C) Three-dimensional confocal micrographs of STIM1/2 DKO MEF cells expressing the indicated mCherry-STIM1 construct (red) and infected for 24 h with C. trachomatis expressing GFP constitutively (green) and IncS_Ct_-3×FLAG under the control of the aTc-inducible promoter for 24 h, in the absence (−aTc) or presence of aTc (+aTc). Scale bar, 5 μm. (D) Quantification of inclusion association (AU) of the indicated mCh-STIM1 constructs when IncS_Ct_ expression is induced (+aTc, black dots) or not (−aTc, gray dots), as in panel C. Each dot represents one inclusion. *n* = 31 inclusions. Unpaired *t* test, ***, *P* < 0.0001; nd, none detected. (E) Coimmunoprecipitation (IP) of IncS_Ct_-3×FLAG from lysates of HEK293 cells expressing GFP or GFP-STIM1 (234–535) and infected for 24 h with C. trachomatis expressing IncS_Ct_-3×FLAG under the control of an aTc-inducible promoter in the absence (−aTc) or the presence (+aTc) of aTc. (F) Three-dimensional confocal micrographs of STIM1/2 DKO MEF cells expressing GFP-STIM1 (234–535) (green) infected for 24 h with a C. trachomatis strain expressing mCherry constitutively (red) and IncS_Ct_-3×FLAG under the control of an aTc-inducible promoter, in the absence (−aTc, upper panels) or the presence (+aTc, lower panels) of aTc. The merge is shown on the right. Scale bar, 5 μm. (G) Quantification of GFP-STIM1 (234–535) association with the inclusion when IncS_Ct_ expression is induced (+aTc, black dots) or not (−aTc, gray dots) in arbitrary units (AU), as in panel F. Each dot represents one inclusion. *n *= 44 inclusions. Unpaired *t* test, ***, *P* < 0.0001.

We next sought to identify the minimal domain of STIM1 required for the STIM1-IncS_Ct_ interaction. STIM1 is a multidomain protein with the N terminus residing in the ER lumen and the C terminus facing the cytosol (28). A series of mCherry-tagged truncated STIM1 constructs, previously described (26) and presented in Fig. 2A, were assessed for their ability to interact with IncS_Ct_ in co-IP experiments. HEK293 cells expressing mCherry-STIM1 constructs, full length (1–685) or truncated in their C-terminal region (1–535 or 1–246), were infected with a wild-type C. trachomatis strain expressing GFP constitutively and IncS_Ct_-3×FLAG under the control of the aTc-inducible promoter (Fig. S1C and D), in the presence or absence of aTc. Lysates were immunoprecipitated, using anti-FLAG-conjugated beads. Total lysates and IP samples were analyzed by Western blotting using anti-mCherry antibodies, to detect mCherry-STIM1, and anti-FLAG antibodies, to detect IncS_Ct_ (Fig. 2B). Full-length mCherry-STIM1 (1–685) and the construct with the deletion of the Ser/Pro-rich (S/P) region and poly(Lys)-rich (K) region (1–535) successfully interacted with IncS_Ct_-3×FLAG, while the mCherry-STIM1 construct truncated for the cytosolic domains (1–246) did not. This result prompted us to test an internal deletion of the region located between amino acids (aa) 253 and 535 (Δ253–535). The corresponding mCh-STIM1 construct failed to immunoprecipitate IncS_Ct_, indicating that the region between aa 253 and 535 is necessary for STIM1-IncS_Ct_ interaction.

We next evaluated the IncS_Ct_-dependent recruitment of the above-mentioned truncated mCh-STIM1 constructs to the inclusion by confocal microscopy (Fig. 2C). To rule out any possible contribution of endogenous STIM1, or its STIM2 homologue (30), we used mouse embryonic fibroblast (MEF) cells knocked out for both STIM1 and STIM2 (STIM1/2 DKO) (31). STIM1/2 DKO MEFs expressing full-length (1–685), 1–535, 1–246, or Δ253–535 mCh-STIM1 were infected with C. trachomatis expressing GFP constitutively and IncS_Ct_-3×FLAG under the control of the aTc-inducible promoter, in the absence or presence of aTc. The samples were fixed 24 h p.i. and analyzed by confocal microscopy. As observed in HeLa cells, induction of IncS_Ct_ expression led to an apparent and significant increase of full-length STIM1 recruitment to the inclusion membrane (Fig. 2C and D, 1–685). A similar result was observed with STIM1 lacking the S/P and poly(K) regions (Fig. 2C and D, 1–535). However, and in agreement with the co-IP results (Fig. 2B), IncS_Ct_ failed to recruit STIM1 lacking the cytosolic domain, or internally truncated for the 253–535 region, to the inclusion membrane (Fig. 2C and D, 1–246 and Δ253–535, respectively). Altogether, these results indicate that the region located between aa 253 and 535 of STIM1 is necessary for IncS_Ct_-STIM1 interaction at the inclusion membrane.

We next investigated the sufficiency of the 253–535 region in the IncS_Ct_-STIM1 interaction. We first generated a translational fusion between GFP and the 234–535 region of STIM1 (GFP-STIM1_234–535_) and tested whether this construct was sufficient to interact with IncS_Ct_ in co-IP experiments. HEK293 cells expressing GFP or GFP-STIM1_234–535_ were infected, or not, with C. trachomatis encoding the inducible IncS_Ct_-3×FLAG construct, in the presence or absence of aTc. Lysates were immunoprecipitated using anti-FLAG-conjugated beads. Total lysates and IP samples were analyzed by Western blotting using anti-GFP antibodies, to detect GFP or GFP-STIM1_234–535_, and anti-FLAG antibodies, to detect IncS_Ct_ (Fig. 2E). In contrast to GFP alone, GFP-STIM1_234–535_ was successfully immunoprecipitated upon induction of IncS_Ct_ expression (Fig. 2E, last lane, IP-FLAG blot).

We next evaluated the ability of IncS_Ct_ to recruit STIM1_234–535_ to the inclusion membrane. STIM1/2 DKO MEFs cells expressing GFP-STIM1_234–535_ were infected with C. trachomatis expressing mCherry constitutively and IncS_Ct_-3×FLAG under the control of the aTc-inducible promoter, in the absence or in the presence of aTc. The samples were fixed 24 h p.i. and analyzed by confocal microscopy (Fig. 2F). In the absence of IncS_Ct_ induction, STIM1_234–535_ was not detected at the inclusion (Fig. 2F, top panels). In contrast, in the presence of aTc, STIM1_234–535_ localized to inclusion (Fig. 2F, bottom panels). Quantification confirmed the IncS_Ct_-dependent recruitment of STIM1_234–535_ to the inclusion (Fig. 2G).

Altogether, these results indicate that the region encompassing aa 234 to 535 of STIM1 is necessary and sufficient for the IncS_Ct_-dependent recruitment of STIM1 to the inclusion.

### The Chlamydia muridarum IncS homologue does not interact with STIM1.

**FIG 3:**
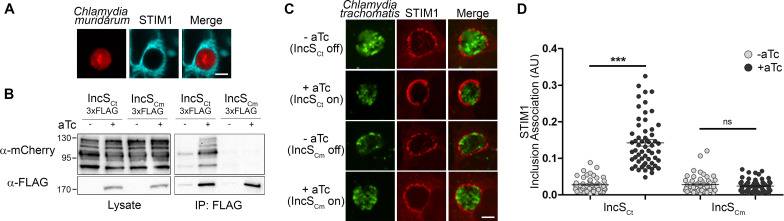
The C. muridarum IncS homologue does not interact with STIM1. (A) Single-plane confocal micrographs of HeLa cells expressing CFP-STIM1 (blue) and infected for 24 h with C. muridarum expressing mCherry constitutively (red). The merge is shown on the right. Scale bar, 5 μm. (B) Coimmunoprecipitation (IP) of IncS_Ct_-3×FLAG or IncS_Cm_-3×FLAG from lysates of HeLa cells expressing mCherry-STIM1 and infected for 24 h with C. trachomatis expressing IncS_Ct_-3×FLAG or IncS_Cm_-3×FLAG under the control of an aTc-inducible promoter in the presence (+aTc) or absence (−aTc) of aTc. Numbers at left are molecular masses in kilodaltons. (C) Three-dimensional confocal micrographs of STIM1/2 DKO MEF cells expressing mCherry-STIM1 (red) and infected for 24 h with C. trachomatis expressing GFP constitutively (green) and IncS_Ct_-3×FLAG or IncS_Cm_-3×FLAG under an aTc-inducible promoter, in the absence (−aTc) or presence (+aTc) of aTc. The merge is shown on the right. Scale bar, 5 μm. (D) Quantification of inclusion association (AU) of mCh-STIM1 when IncS_Ct_ or IncS_Cm_ is induced (+aTc) or not (−aTc), as in panel C. Each dot represents one inclusion. *n* = 57 inclusions. Unpaired *t* test, ***, *P* < 0.0001; ns, not significant.

To determine if the IncS_Ct_-dependent recruitment of STIM1 to the inclusion membrane is conserved in other Chlamydia species encoding IncS homologues, we investigated STIM1 association with C. muridarum inclusions. Capitalizing on the fact that human and murine STIM1 share 96.79% identity and that the 253–535 region important for IncS interaction is 100% identical between the two species, human STIM1 was used for these studies. HeLa cells expressing CFP-STIM1 were infected with C. muridarum expressing mCherry constitutively and analyzed by confocal microscopy at 24 h p.i. To our surprise, STIM1 was not recruited to C. muridarum inclusions (Fig. 3A). This result prompted us to evaluate whether the C. muridarum IncS homologue (TC0424, referred to as IncS_Cm_) was capable of interacting with STIM1. We generated a C. trachomatis strain expressing GFP constitutively and IncS_Cm_-3×FLAG under the control of the aTc-inducible promoter. Lysates from HeLa cells expressing mCherry-STIM1 and infected with C. trachomatis strains expressing inducible IncS_Ct_- or IncS_Cm_-3×FLAG, in the absence or presence of aTc, were immunoprecipitated using anti-FLAG-conjugated beads. Total lysates and IP samples were analyzed by Western blotting using anti-mCherry antibodies, to detect mCherry-STIM1, and anti-FLAG antibodies, to detect IncS_Ct_ or IncS_Cm_ (Fig. 3B). As opposed to IncS_Ct_, IncS_Cm_ failed to immunoprecipitate with STIM1 (Fig. 3B, compare lanes 2 and 4 of IP:FLAG blot). To further validate the lack of IncS_Cm_-STIM1 interaction, STIM1/2 DKO MEF cells expressing mCherry-STIM1 were infected with C. trachomatis strains expressing inducible IncS_Ct_- or IncS_Cm_-3×FLAG, in the absence or presence of aTc. The samples were fixed 24 h p.i. and analyzed by confocal microscopy (Fig. 3C). As shown in Fig. 1C and D and Fig. 2C and D, IncS_Ct_ expression led to a significant increase in STIM1 association with the inclusion (Fig. 3C, upper two panels, and Fig. 3D, IncS_Ct_). In contrast, IncS_Cm_ expression did not result in a significant increase in STIM1 recruitment to the inclusion membrane (Fig. 3C, lower two panels, and Fig. 3D, IncS_Cm_). Altogether, these results indicate that STIM1 does not associate with C. muridarum inclusions and that IncS_Cm_ does not interact with STIM1.

### IncS_Ct_ is necessary for STIM1 association with the C. trachomatis inclusion.

**FIG 4:**
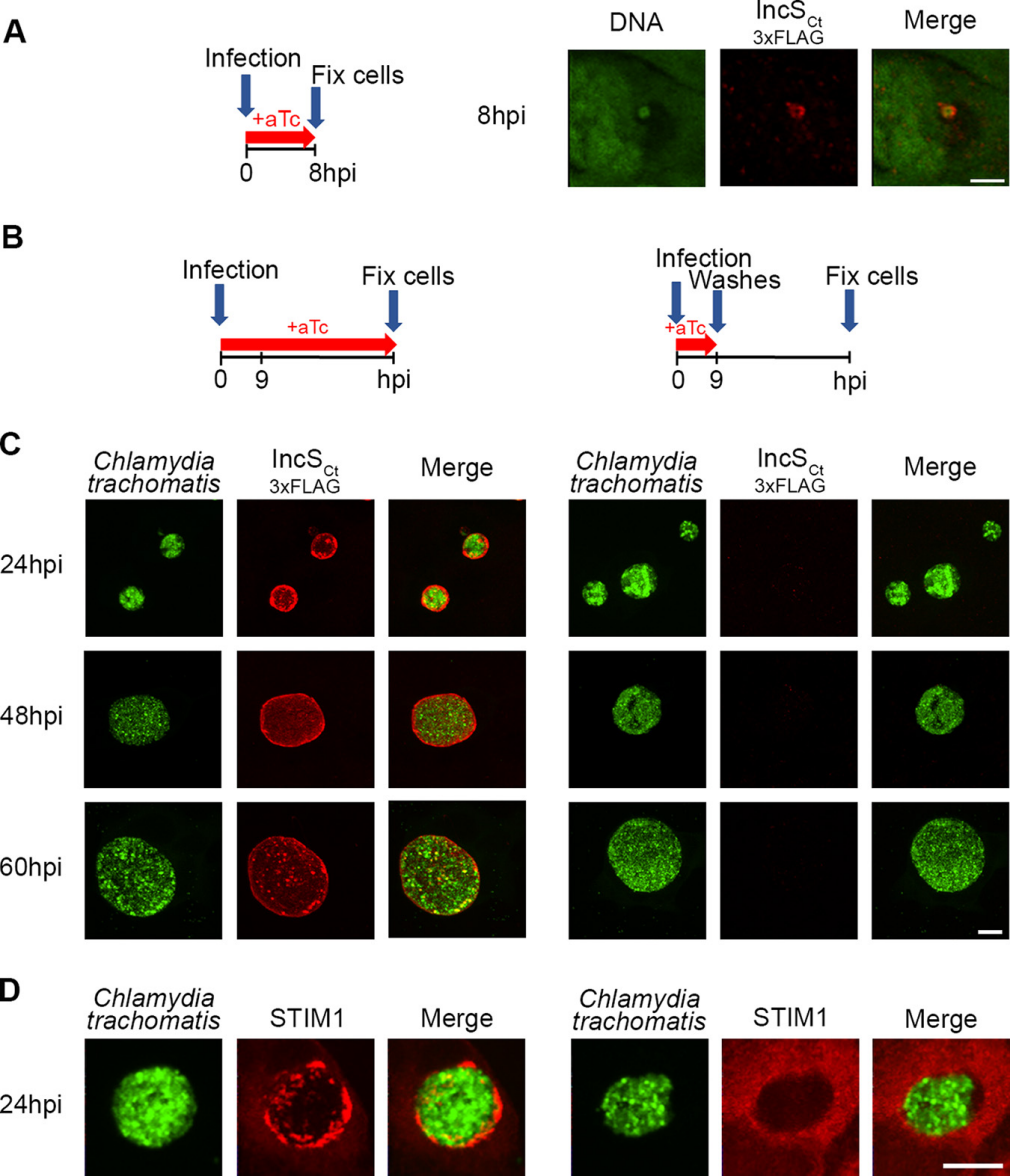
IncS_Ct_ is necessary for STIM1 association with the C. trachomatis inclusion. (A) (Left) Infection scheme. (Right) Single-plane confocal micrographs of HeLa cells infected for 8 h with a C. trachomatis *incS_Ct_* conditional mutant expressing IncS_Ct_-3×FLAG (red) under the control of an aTc-inducible promoter in the presence of aTc. DNA is in green. The merge is on the right. Scale bar, 5 μm. (B) Infection scheme for panels C and D. (C) Three-dimensional confocal micrographs of HeLa cells infected for the indicated times with a C. trachomatis *incS_Ct_* conditional mutant strain expressing GFP constitutively (green) and IncS_Ct_-3×FLAG (red) under the control of an aTc-inducible promoter. aTc was kept for the duration of the whole experiment (left panels) or only for the first 9 h and then removed with intensive washes (right panels). Scale bar, 10 μm. (D) Three-dimensional confocal micrographs of HeLa cells expressing mCh-STIM1 and infected for 24 h with C. trachomatis *incS_Ct_* conditional mutant, as in panel C. Scale bar, 10 μm.

We next investigated whether IncS_Ct_ was necessary for STIM1 association with C. trachomatis inclusion. However, we recently showed that a C. trachomatis *incS_Ct_* mutant does not progress through the early stages of the C. trachomatis developmental cycle (29). To bypass the early developmental defect of the *incS_Ct_* mutant, we capitalized on the fact that the severe growth defect of the C. trachomatis *incS_Ct_* mutant can be complemented by inducing *incS_Ct_* transcription during the first 6 to 8 h p.i. of the developmental cycle (29). Under this condition, we found that IncS_Ct_ is present at the inclusion 8 h p.i. (Fig. 4A). To determine if stopping *incS_Ct_* transcription, shortly after the early stage of the developmental cycle, would result in loss of IncS_Ct_ at the inclusion at later time points, HeLa cells were infected with the C. trachomatis *incS_Ct_* mutant in the presence of aTc for the entire duration of the experiment or for only the first 9 h (Fig. 4B and C). The samples were fixed at 24 h, 48 h, and 60 h p.i., stained with anti-FLAG antibodies, and analyzed by confocal microscopy. Full complementation of the C. trachomatis *incS_Ct_* mutant resulted in IncS_Ct_-positive inclusions that increased in size over time (Fig. 4C, left panels). Addition of aTc in the early stages of the development cycle also resulted in inclusion growth over time, indicating successful complementation of the early developmental defect. However, while inclusions were IncS_Ct_ positive prior to aTc removal (Fig. 4A), IncS_Ct_ was no longer detectable at later time points, indicating that temporal complementation of IncS expression was successfully achieved (Fig. 4C, right panels). To next determine whether IncS_Ct_ was essential for STIM1 recruitment to C. trachomatis inclusions, HeLa cells expressing mCherry-STIM1 were infected with the C. trachomatis *incS_Ct_* mutant under the conditions described above to achieve temporal complementation. The samples were fixed at 24 h p.i. and analyzed by confocal microscopy. Under complementation during the entire developmental cycle, IncS_Ct_ expression led to STIM1 association with the inclusion (Fig. 4D, left panels). However, preventing IncS_Ct_ inclusion localization beyond the early stages of the developmental cycle resulted in STIM1-negative inclusion (Fig. 4D, right panels). Altogether, these results indicate that STIM1 is not recruited to C. trachomatis inclusions in the absence of IncS_Ct_.

### Characterization of the allelic replacement of *incS_Ct_* with *incS_Cm_* at the C. trachomatis *incS_Ct_* locus.

**FIG 5:**
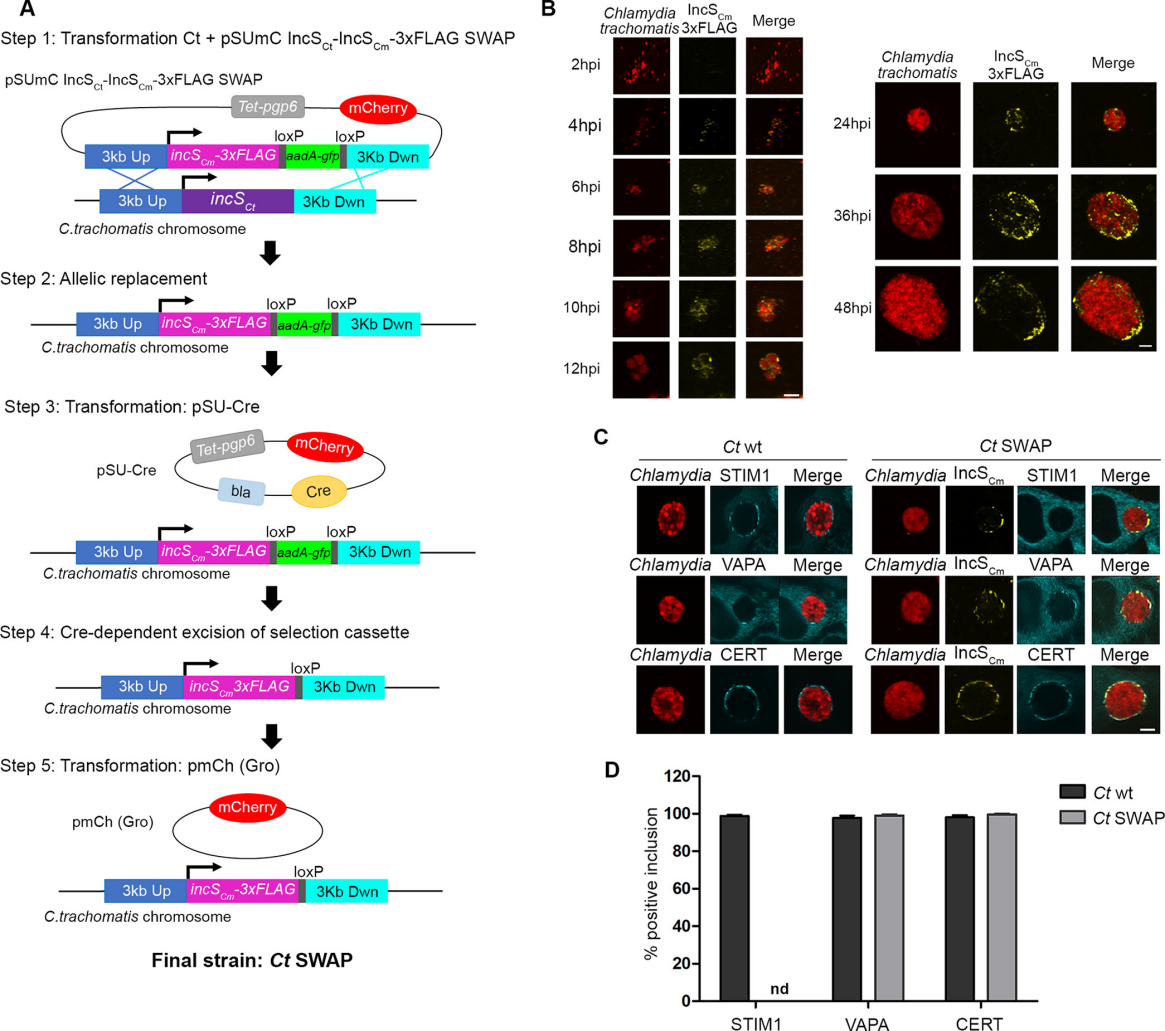
Characterization of the allelic replacement of *incS_Ct_* with *incS_Cm_* at the C. trachomatis *incS_Ct_* locus. (A) Cartoon depicting the strategy employed to generate the C. trachomatis SWAP strain in which the *incS_Ct_* ORF was replaced with the *incS_Cm_* ORF using FLAEM. (Step 1) Transformation of wild-type C. trachomatis, containing an intact chromosomal *incS_Ct_* ORF (purple), with the pSUmC IncS_Ct_-IncS_Cm_-3×FLAG SWAP plasmid harboring (i) ~3 kb of homology sequence directly upstream and downstream of the *incS_Ct_* ORF (3 kb Up [blue] with the black arrow indicating the *incS_Ct_* promoter and 3 kb Dwn [cyan]), flanking the *incS_Cm_* ORF fused to 3×FLAG tag (pink) and the *aadA-gfp* selection cassette (green) flanked by two loxP sites (gray), (ii) mCherry (red), and (iii) the plasmid maintenance *pgp6* ORF under the control of the aTc-inducible promoter (light gray). (Step 2) Upon allelic replacement by two recombination events, the chromosomal *incS_Ct_* ORF is replaced by *incS_Cm_*-3×FLAG + *aadA-gfp* selection cassette flanked by the two loxP sites. (Step 3) Transformation of the strain from step 2 with pSU-Cre, a plasmid encoding (i) the Cre recombinase (orange), (ii) mCherry, (iii) the plasmid maintenance *pgp6* ORF under the control of the aTc-inducible promoter (light gray), or (iv) beta-lactamase (*bla*, light blue). (Step 4) Cre-mediated excision of *aadA-gfp* cassette recombination leads to a single loxP site (gray) between *incS_Cm_*-3×FLAG (pink) and the downstream region (cyan). (Step 5) Transformation of the strain obtained in step 4 with a plasmid carrying mCherry (red). The final strain is named C. trachomatis SWAP. (B) Confocal micrographs of HeLa cells infected for the indicated times with the C. trachomatis SWAP strain expressing mCherry (red) constitutively and IncS_Cm_-3×FLAG under the *incS_Ct_* endogenous promoter (yellow) at an MOI of 10 (left panels, single planes) or at an MOI of 1 (right panels, 3-dimensional reconstruction). Merged images are shown on the right. Scale bar, 5 μm. (C) Single-plane confocal micrographs of HeLa cells expressing CFP-STIM1, CFP-VAPA, or CFP-CERT (blue) infected for 24 h with wild-type C. trachomatis (*Ct* wt) or the C. trachomatis SWAP strain (*Ct* SWAP) expressing mCherry (red) constitutively and IncS_Cm_-3×FLAG under the control of the *incS_Ct_* endogenous promoter (yellow). Scale bar, 5 μm. (D) Quantification of the percentage of inclusions that recruits STIM1, VAPA, or CERT to the inclusion membrane, as in panel C. *n* = 100 inclusions. nd, nondetected.

To further explore the respective properties of *incS_Ct_* and *incS_Cm_*, we set out to generate a C. trachomatis strain expressing IncS_Cm_ instead of IncS_Ct_. To this end, we designed a *f*loxed cassette *a*llelic *e*xchange *m*utagenesis (FLAEM) (32)-based strategy (Fig. 5A and Materials and Methods) to engineer the C. trachomatis chromosome such that the *incS_Ct_* open reading frame (ORF) was replaced with a 3×FLAG-tagged *incS_Cm_* allele, yielding a C. trachomatis strain encoding IncS_Cm_-3×FLAG under the control of the native *incS_Ct_* promoter at the C. trachomatis *incS_Ct_* locus. The allelic replacement of *incS_Ct_* with *incS_Cm_* and the excision of the *aadA-gfp* selection cassette were confirmed by PCR (Fig. S2). The corresponding C. trachomatis strain, which also expresses mCherry constitutively from a plasmid, is referred to as the C. trachomatis SWAP strain.

10.1128/msphere.00003-23.2 FIG S2 Validation of C. trachomatis SWAP strain. (A) Schematic representation of C. trachomatis SWAP strain chromosome configuration before (−Cre) and after (+Cre) Cre recombinase excision of the *aadA-gfp* selection cassette. Blue, ~3-kb homology sequence upstream of the *incS_Ct_* ORF (3kb Up) with the black arrow indicating the *incS_Ct_* promoter; cyan, ~3-kb homology sequence downstream of the *incS_Ct_* ORF (3kb Dwn); pink, *incS_Cm_* ORF fused to 3×FLAG tag; green, the *aadA-gfp* selection cassette; gray, loxP sites. The arrows annotated P1, P2, P3, and P4 indicate the positions of the primers used for PCR validation shown in panels B and C. (B and C) DNA gels of PCR products generated using the following combination of primers (as described in panel A) and genomic DNA from the SWAP strain before (−Cre) and after (+Cre) Cre-lox-mediated excision of the *aadA-gfp* selection cassette (+Cre). −Cre/P1P2 (B, lane 2), +Cre/P1P2 (B, lane 3), −Cre/P3P4 (C, lane 1) +Cre/P3P4 (C, lane 2). The size ladder is shown in lane 1 (B) and lane 3 (C). (D) The *incS_Ct_* promoter-*incS_Cm_* DNA junction and the excision of the *aadA-gfp* cassette were confirmed by Sanger sequencing of the fragment amplified from the PCR product generated with (+Cre) genomic DNA and the P1 and P2 primers. The color key is the same as in panel A. Download FIG S2, TIF file, 2.6 MB. Copyright © 2023 Cortina and Derré. 2023 Cortina and Derré. https://creativecommons.org/licenses/by/4.0/ This content is distributed under the terms of the Creative Commons Attribution 4.0 International license.

To evaluate the timing of expression of IncS_Cm_ from the endogenous *incS_Ct_* promoter, HeLa cells were infected with the C. trachomatis SWAP strain, fixed at the indicated time points, stained with anti-FLAG antibodies, and analyzed by confocal microscopy (Fig. 5B). IncS_Cm_-3×FLAG was detected in association with the mCherry-positive inclusions as early as 4 h p.i., and throughout the developmental cycle until 48 h p.i. (the latest time point tested), indicating proper expression and inclusion localization of IncS_Cm_ from the *incS_Ct_* endogenous promoter. To determine if STIM1 was recruited to C. trachomatis inclusions displaying IncS_Cm_ instead of IncS_Ct_, HeLa cells expressing CFP-STIM1 were infected with wild-type C. trachomatis expressing mCherry constitutively or with the C. trachomatis SWAP strain. The samples were fixed 24 h p.i., stained with anti-FLAG antibodies, and analyzed by confocal microscopy (Fig. 5C). As previously observed, wild-type C. trachomatis inclusions displayed small STIM1 patches (Fig. 5C, upper left panels). In contrast, despite the presence of IncS_Cm_ at the inclusion, C. trachomatis SWAP inclusions were negative for STIM1 (Fig. 5C, upper right panels). Quantification of the percentage of STIM1 inclusions confirmed these observations (Fig. 5D, STIM1). To determine if C. trachomatis SWAP inclusions were also defective for recruitment of other known components of the ER-inclusion MCS, we evaluated the recruitment of VAPA and CERT. In contrast to the lack of STIM1 association, C. trachomatis SWAP inclusions displayed small VAPA and CERT patches, similar to wild-type inclusions (Fig. 5C, middle and upper panels, respectively; Fig. 5D, quantification), indicating the specific lack of STIM1 association with C. trachomatis SWAP inclusions.

Altogether, these results indicate that IncS_Ct_ is necessary for STIM1 recruitment to C. trachomatis inclusions and that expression of IncS_Cm_ cannot complement this phenotype. This is in contrast with the interchangeability of IncS_Ct_ and IncS_Cm_ for progression through the early stages of the developmental cycle, suggesting shared and distinct functions for the C. trachomatis and C. muridarum IncS homologues.

### Lack of IncS_Ct_ results in inclusion lysis at late stages of the developmental cycle.

**FIG 6:**
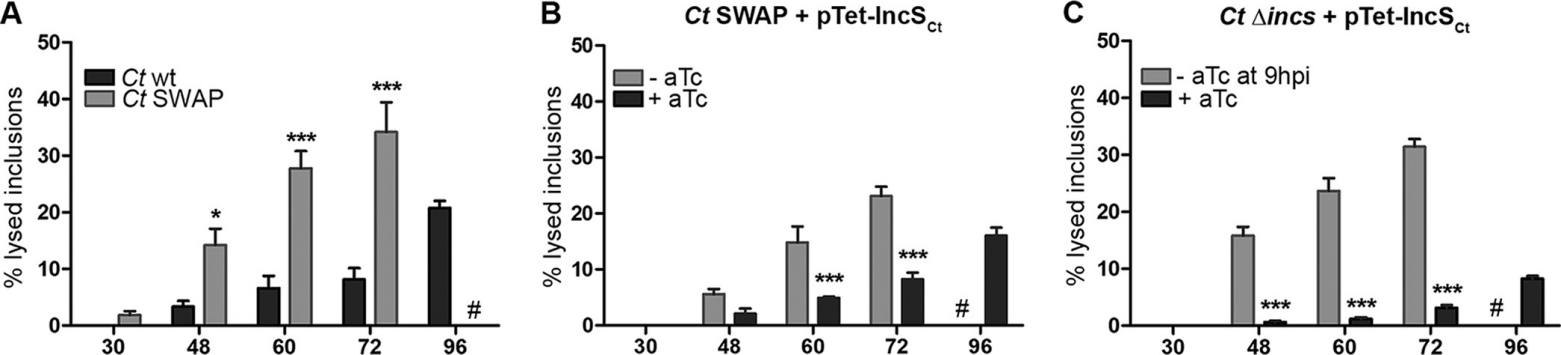
Lack of IncS_Ct_ results in inclusion lysis at late stages of the developmental cycle. Quantification of the percentage of lysed inclusions at the indicated time postinfection in HeLa cells infected with wild-type C. trachomatis (*Ct* wt) (A), the C. trachomatis SWAP strain (*Ct* SWAP) (A), the C. trachomatis SWAP strain harboring pTet-IncS_Ct_ (*Ct* SWAP+pTet-IncS_Ct_) in the presence (+aTc) or absence (−aTc) of aTc (B), or C. trachomatis *incS_Ct_* conditional mutant (Δ*incS_Ct_*+pTet-IncS_Ct_) in the presence of aTc at all times (+aTc) or for the first 9 h (−aTc at 9 hpi) (C). *n* = 150 inclusions. Two-way analysis of variance (ANOVA), Bonferroni posttest. *, *P* < 0.05; ***, *P* < 0.001. #, not quantified due to excessive lysis.

STIM1 depletion does not affect the production of C. trachomatis infectious progeny at 48 h p.i. (26, 27); however, exit via extrusion is reduced (27). To investigate the potential role of IncS_Ct_ in C. trachomatis exit via extrusion, we first attempted to measure extrusion of the C. trachomatis SWAP strain. To this end, HeLa cells were infected with wild-type C. trachomatis or the SWAP strain and the production of infectious progeny or extrusion was analyzed at 48 h p.i. The C. trachomatis SWAP strain produced similar amounts of infectious particles as wild-type C. trachomatis (Fig. S3A). However, cells infected with the C. trachomatis SWAP strain displayed a high proportion of lysed inclusions, usually not observed at 48 h p.i., that prevented us from quantifying extrusion defects. To further characterize the timing of inclusion lysis of the C. trachomatis SWAP strain, HeLa cells infected with wild-type C. trachomatis or the C. trachomatis SWAP strain expressing mCherry were monitored live at different time points p.i. (30 h, 48 h, 60 h, 72 h, and 96 h p.i.). At each time point the percentage of inclusions that had undergone lysis was quantified (Fig. 6A). Compared to the wild-type strain, a significant increase in the percentage of lysed inclusions was observed starting at 48 h p.i. with the C. trachomatis SWAP strain.

10.1128/msphere.00003-23.3 FIG S3 Characterization of the C. trachomatis SWAP+pTet-IncS_Ct_ strain. (A) Fold growth of the wild-type C. trachomatis (*Ct* wt), the C. trachomatis SWAP strain (*Ct* SWAP), and the C. trachomatis SWAP strain harboring pTet-IncS_Ct_ (*Ct* SWAP+pTet-IncS_Ct_) in the presence (+aTc) or absence (−aTc) of aTc, in HeLa cells at 48 h. (B) Three-dimensional confocal images of HeLa cells infected for 24 h with the C. trachomatis SWAP strain expressing mCherry (red) constitutively, IncS_Cm_-3×FLAG under the *incS_Ct_* endogenous promoter (yellow), and IncS_Ct_-HA (blue) under the control of an aTc-inducible promoter, in the absence (−aTc) or the presence (+aTc) of aTc. Scale bar, 5 μm. Download FIG S3, TIF file, 2.5 MB. Copyright © 2023 Cortina and Derré. 2023 Cortina and Derré. https://creativecommons.org/licenses/by/4.0/ This content is distributed under the terms of the Creative Commons Attribution 4.0 International license.

To determine if this phenotype was due to the lack of IncS_Ct_, we generated a C. trachomatis SWAP strain harboring a complementation plasmid expressing mCherry constitutively and IncS_Ct_ fused to a hemagglutinin (HA) tag under the aTc-inducible promoter. The strain is referred to as C. trachomatis SWAP+pTet-IncS_Ct_. The inducible expression and inclusion localization of IncS_Ct_-HA was confirmed by immunofluorescence (IF) (Fig. S3B); the strain produced similar numbers of infectious progeny in the presence or absence of aTc as the wild-type C. trachomatis and original C. trachomatis SWAP strains (Fig. S3A). We next infected HeLa cells with the C. trachomatis SWAP+pTet-IncS_Ct_ strain in the absence or presence of aTc and monitored inclusion lysis over time (Fig. 6B). In the absence of aTc, the C. trachomatis SWAP+pTetIncS_Ct_ strain displayed a significant increase of inclusion lysis in the late stage of the developmental cycle, as observed for the original SWAP strain (Fig. 6B, gray bars). In comparison, aTc induction of the *incS_Ct_* complementation allele resulted in a significant decrease in lysis (Fig. 6B, black bars). These results indicate that the increase in inclusion lysis of the C. trachomatis SWAP strain is due to the absence of IncS_Ct_, rather than the expression of IncS_Cm_.

To further confirm our conclusion, we analyzed the timing of inclusion lysis of the C. trachomatis *incS_Ct_* mutant upon temporal complementation, as described in Fig. 4. HeLa cells were infected with the C. trachomatis *incS_Ct_* conditional mutant, in the presence of aTc during the entire developmental cycle or only during the first 9 h. Inclusion lysis was monitored live and quantified at the indicated time points (Fig. 6C). As observed with the C. trachomatis SWAP strain, in the absence of IncS_Ct_ at the inclusion membrane past the early stages in the developmental cycle, a significant increase in inclusion lysis was observed 48 h p.i. and onward (Fig. 6C, gray bars). This phenotype was complemented by expression of IncS_Ct_ throughout the developmental cycle (Fig. 6C, black bars).

Altogether, these results indicate that the absence of IncS_Ct_ on the C. trachomatis inclusion results in increased inclusion lysis in the late stages of the developmental cycle.

## DISCUSSION

### Advances in Chlamydia genetic manipulation to characterize effector function and function conservation across species.

The identification and characterization of Chlamydia effector proteins have been significantly impaired by the genetic intractability of the organism. In the past years, the Fluorescence-Reported Allelic Exchange Mutagenesis (FRAEM)/FLAEM methodology has been instrumental in achieving targeted gene inactivation and *cis* complementation (33–35). Additionally, we recently reported a blueprint for the FRAEM-based generation of conditional mutants in essential genes, such as genes critical for progression through the early stages of the developmental cycle, like *incS* (29). We showed that induction of a complementation allele exclusively during the first 8 h of the developmental cycle was sufficient to complement the early developmental defect of the C. trachomatis *incS_Ct_* mutant (29). Here, we show that temporal complementation, exclusively in the early stages of the developmental cycle, rescued the early developmental defect of the C. trachomatis *incS_Ct_* mutant, while revealing and allowing us to investigate an additional role of IncS_Ct_ during the late stages of the developmental cycle. While the success of this powerful approach highly depends on the stability of the effector protein of interest, it will most certainly benefit the field when dissecting the role of effector proteins at a specific stage of the developmental cycle. Similar to IncS_Ct_, which appears to play multiple roles both early and late, we anticipate that, given the reduced size of the Chlamydia genome (36), effector proteins displaying multiple functions may be common in Chlamydia.

Another significant advance in Chlamydia genetic manipulation presented here is the use of the FLAEM methodology to specifically and precisely engineer the C. trachomatis chromosome to achieve the replacement of a specific ORF with a different ORF. Replacing the *incS_Ct_* ORF with its C. muridarum homologue, in the C. trachomatis chromosome, was instrumental in investigating potential conservation of IncS function between C. trachomatis and C. muridarum. While IncS_Ct_ and IncS_Cm_ shared a conserved function in the early stages of the developmental cycle, IncS_Cm_ failed to rescue the late lysis defect, pointing toward different functions later in the developmental cycle, which could be attributed to the fact that IncS_Ct_ and IncS_Cm_ share only 60% and 70% identity and similarity, respectively (see Fig. S5 in the supplemental material). Overall, the differences in primary amino acid sequence between IncS_Ct_ and IncS_Cm_ are restricted to individual amino acids evenly distributed throughout the IncS proteins. However, a few motifs consisting of 5 to 10 aa are poorly conserved and will be the focus of future studies. Overall, our results highlight that effector function may not be fully conserved among Chlamydia species, which is an important consideration as the field moves forward, especially when C. muridarum should be the default species for *in vivo* studies, because of the inability of C. trachomatis to establish long-lasting infection due to failure to counteract the host interferon gamma response (37).

10.1128/msphere.00003-23.5 FIG S5 Primary amino acid sequence alignment of IncS_Ct_ and IncS_Cm_. IncS_Ct_ and IncS_Cm_ proteins were aligned with Clustal Omega software. The asterisk indicates positions which have a single, fully conserved residue; the colon indicates conservation between groups of strongly similar properties, scoring >0.5 in the Gonnet PAM 250 matrix; the period indicates conservation between groups of weakly similar properties, scoring ≤0.5 in the Gonnet PAM 250 matrix. The black rectangles denote IncS putative transmembrane domains. The amino acid position is indicated to the right. Download FIG S5, TIF file, 3.1 MB. Copyright © 2023 Cortina and Derré. 2023 Cortina and Derré. https://creativecommons.org/licenses/by/4.0/ This content is distributed under the terms of the Creative Commons Attribution 4.0 International license.

Our FLAEM-based chromosome engineering strategy was also designed for expression of a tagged version of the protein of interest, allowing us to track expression of effector proteins from their endogenous promoter at the locus, thus eliminating any artifact of overexpression and/or improper timing of expression. The methodology described here could be easily applied for further manipulation of the Chlamydia genome, such as introducing a point mutation(s) in promoters or coding regions of interest. Furthermore, while the original FRAEM/FLAEM tools were restricted to C. trachomatis (33, 34), one can envision that the recent development of a minimal replicon for allelic replacement in other Chlamydia species, such as C. muridarum (38), will be instrumental in applying the method described here to investigate effector function in C. muridarum.

### IncS_Ct_-STIM1 interaction at ER-inclusion MCS.

Through gain- and loss-of-function studies, we showed that the C. trachomatis inclusion membrane protein IncS_Ct_ interacts with, recruits, and colocalizes with STIM1 at the inclusion, thus identifying IncS_Ct_ as a novel component of ER-inclusion MCS. Identification of the IncS_Ct_-STIM1 complex is in line with the current model that ER-inclusion MCS localized protein complexes are composed of specific inclusion membrane proteins that each interact with a defined set of host factors (17, 24, 25).

While the respective role of the IncD-CERT-VAP and IncV-VAP complexes in lipid acquisition and ER-inclusion tethering is fairly well established (17, 23–25), the role of the IncS_Ct_-STIM1 complex remains elusive. The concomitant expression and inclusion localization of IncS_Ct_ with the recruitment of STIM1 to the inclusion, starting early and lasting throughout the C. trachomatis developmental cycle (26), are in line with a shared function for IncS_Ct_ and STIM1. However, a functional connection has yet to be established. Depletion of STIM1 did not result in, nor did it rescue, the early developmental defect or the late inclusion lysis phenotype observed with the *incS_Ct_* mutant, suggesting that STIM1 is not involved in these processes (Fig. S4). Moreover, because the increased inclusion lysis observed in the absence of IncS_Ct_ prevented assaying for a potential extrusion defect, it remains to be determined whether the extrusion defect observed upon STIM1 depletion (27) is due to a lack of IncS_Ct_-STIM1 interaction at ER-inclusion MCS.

10.1128/msphere.00003-23.4 FIG S4 Depletion of STIM1 does not result in, nor rescue, the late inclusion lysis phenotype observed with strains lacking *incS_Ct_*. Quantification of the percentage of lysed inclusions at the indicated time postinfection in wild-type and STIM1/2 DKO MEFs infected with wild-type C. trachomatis (*Ct* wt) (A) and the C. trachomatis SWAP strain harboring pTet-IncS_Ct_ (*Ct* SWAP+pTet-IncS_Ct_) in the presence (+aTc) (B) or absence (−aTc) of aTc (C). *n* = 150 inclusions. Two-way analysis of variance, Bonferroni posttest. Download FIG S4, TIF file, 3.5 MB. Copyright © 2023 Cortina and Derré. 2023 Cortina and Derré. https://creativecommons.org/licenses/by/4.0/ This content is distributed under the terms of the Creative Commons Attribution 4.0 International license.

A recent study by Chamberlain et al. may help shed light on the function of IncS_Ct_-STIM1 in midcycle (39). More specifically, the authors reported impaired SOCE-dependent NFAT (nuclear factor of activated T cells) nuclear translocation in C. trachomatis-infected cells and proposed that STIM1 retention at ER-inclusion MCS, instead of proper relocalization to ER-PM MCS, may explain this phenotype and consequently lead to the downregulation of chemokine and cytokines downstream of NFAT signaling. Based on these observations, future studies focused on NFAT-dependent signaling pathways in cells infected with the *incS_Ct_* mutant may help shed light on the biological significance of the IncS_Ct_-STIM1 interaction at ER-inclusion MCS.

### Inclusion membrane proteins and inclusion membrane stability.

The inclusion membrane cloaks the bacteria from host surveillance pathways and allows for a safe replicative niche, until completion of the developmental cycle and the release of the infectious progeny. It was proposed that some inclusion membrane proteins play a structural role in maintaining inclusion membrane integrity (15). Moreover, a role in the specific inhibition of innate immune pathways and/or inclusion targeting by components of these pathways is supported by the fact that premature inclusion membrane rupture in the middle stages of the developmental cycle triggers host cell death pathways such as apoptosis, upon infection with C. trachomatis strains lacking CT229/CpoS, CT233/IncC, and CT383 (40–42). IncS_Ct_-negative inclusions were lysed earlier than wild-type inclusions; however, in comparison to strains lacking CT229/CpoS, CT233/IncC, or CT383, lysis occurred much later in the developmental cycle, suggesting that if IncS is also involved in counteracting innate immune defenses, it does so in the late development cycle.

The Inc protein CT0390 has been implicated in the end of the developmental cycle bacterial exit via lysis through the activation of STING (43). The proposed role of STIM1 in retaining STING in the ER, and thereby preventing STING activation, (44), led us to investigate if the IncS_Ct_-STIM1 complex could interfere with activation of the STING pathway to maintain integrity of the inclusion until completion of the developmental cycle. However, genetic and pharmacological inhibition of STING did not rescue the lysis phenotype of IncS_Ct_-negative inclusions (data not shown).

The results presented here are partially in agreement with a study by Dimond et al. proposing that IncS stabilizes the inclusion membrane and prevents host cell death by apoptosis during midcycle (45). However, some discrepancies remain in the timing of inclusion membrane destabilization: midcycle (45) versus late (this study). The study by Dimond et al. (45) relied on C. trachomatis/C. muridarum chimera strains generated by lateral gene transfer, in which large regions of the C. trachomatis chromosome were replaced with regions of the C. muridarum chromosome. A chimera strain (RC826), in which a segment of the C. trachomatis chromosome, including the *incS_Ct_* ORF and some of the immediate downstream sequences, was replaced with the homologous C. muridarum sequence, presented premature inclusion rupture during midcycle, followed by cell death with signs of apoptosis, resulting in a strain that was challenging to amplify. Through backcross experiments, the phenotype of RC826 was linked to the exchange of *incS_Ct_* for *incS_Cm_* and by extension the lack of IncS_Ct_ expression. However, complementation of RC826 by expression of IncS_Ct_ in *trans* was not performed; therefore, the phenotype of the chimera could also be due to IncS_Cm_ expression in the C. trachomatis/C. muridarum chimeric background. The C. trachomatis SWAP strain presented here potentially allows us to settle between these two possibilities. Compared to RC826, the precise replacement of the *incS_Ct_* ORF with the *incS_Cm_* homologue in an intact C. trachomatis genetic background did not result in any inclusion instability until late in the developmental cycle, and this phenotype was complemented by expression of IncS_Ct_ in *trans*. These results suggest that the midcycle inclusion rupture of RC826 may be due to a combinatory effect of the IncS_Ct/Cm_ exchange and of the C. trachomatis/C. muridarum genetic background. Regardless, both strains point toward a role for IncS_Ct_ in inclusion stability.

In future studies, it will be important to consider the proper timing of IncS-dependent inclusion rupture, as it may have significant biological implications relevant to the characterization of the exact molecular mechanism resulting in the lysis of IncS_Ct_-negative inclusions. Based on our results, we favor a role for IncS_Ct_ in maintaining inclusion stability late in the developmental cycle. A non-mutually exclusive hypothesis, potentially in line with a role of IncS_Ct_-STIM1 in exit via extrusion, would be that an increase in inclusion lysis may be reflective of a shift in the balance of bacterial exit toward lysis rather than extrusion (9).

### Conclusion.

Through the use of innovative genetic approaches, we have dissected the species specificity and temporal function of the Chlamydia inclusion protein IncS. Our study revealed that in addition to the previously reported conserved role in the early stages of developmental (29), IncS performs a species-specific role in inclusion stability during the late developmental cycle. Altogether, our results highlight that Inc protein function may display multiple functions depending on the stage of the developmental cycle and may not be fully conserved among species.

## MATERIALS AND METHODS

### Ethics statement.

All genetic manipulations and containment work were approved by the University of Virginia Biosafety Committee and are in compliance with section III-D-1-a of the NIH guidelines for research involving recombinant DNA molecules.

### Cell lines and bacterial strains.

HeLa cells (CCL-2) and HEK293 cells (CRL-1573) were obtained from ATCC. Wild-type and STIM1/2 double-knockout MEF cells were a gift from Masatsugu Oh-hora and were described previously (31). All cell lines were cultured at 37°C with 5% CO_2_ in high-glucose Dulbecco’s modified Eagle’s medium (DMEM; Invitrogen) supplemented with 10% heat-inactivated fetal bovine serum (FBS; Invitrogen). C. trachomatis lymphogranuloma venereum (LGV) type II was obtained from the ATCC (L2/434/Bu VR-902B). C. muridarum Nigg was obtained from Michael Starnbach (Harvard Medical School, Boston, MA). mCherry-expressing C. trachomatis [C. trachomatis mCh(Gro)] and C. muridarum [C. muridarum mCh(Gro)] strains were described previously (46). Chlamydia propagation, infection, and transformation were performed as previously described (22, 29). All Chlamydia strains were plaque purified.

### Cloning.

Restriction enzymes and T4 DNA ligase were obtained from New England BioLabs (Ipswich, MA). PCR was performed using Herculase DNA polymerase (Stratagene). PCR primers were obtained from Integrated DNA Technologies. Primers and cloning strategies are described in Table S1 in the supplemental material and detailed below.

10.1128/msphere.00003-23.6 TABLE S1 Primers and cloning strategies. List of primers and cloning strategies used in this study. Download Table S1, DOCX file, 0.02 MB. Copyright © 2023 Cortina and Derré. 2023 Cortina and Derré. https://creativecommons.org/licenses/by/4.0/ This content is distributed under the terms of the Creative Commons Attribution 4.0 International license.

### Construction of p2TK2_Spec_-SW2 mCh(Gro_L2_) TetR-tetA^P^ IncS_Ct_-3×FLAG *incDEFG* terminator.

DNA fragments corresponding to the *tet* repressor (TetR) and *tetA* promoter (*tetA^P^*) (PCR A) and to the 3×FLAG and the *incDEFG* operon terminator (PCR C) were amplified by PCR from p2TK2-SW2 mCh(Gro) Tet-IncV-3F plasmid (24) using primers TetRSTOP5Kpn and TetAP-IncS Rv and primers 0402FLAG Fw and IncDTerm3Not, respectively. A DNA fragment corresponding to the *ctl0402* (*incS_Ct_*) ORF (PCR B) was amplified from C. trachomatis L2 genomic DNA by PCR using primers TetAP-IncS Fw and 0402 FLAG Rv. A DNA fragment corresponding to TetR-tetA^P^ IncS_Ct_ 3×FLAG *incDEFG* terminator (PCR D) was amplified by overlapping PCR using PCR A, B, and C as the template and primers TetRSTOP5Kpn and IncDTerm3Not. PCR D was cloned into the KpnI/NotI sites of p2TK2Spec-SW2 mCh(Gro_L2_) (46).

### Construction of p2TK2_Spec_-SW2 GFP(nmP) TetR-tetA^P^ IncS_Ct_-3×FLAG *incDEFG* terminator.

A DNA fragment (PCR E) corresponding to Neisseria meningitidis promoter-GFP (nmP-GFP) was amplified by PCR from pGFP::SW2 (47) with primers AgeI nmP Prom Fw and RSGFP TAA AgeI Rv and cloned into the AgeI site of the plasmid p2TK2_Spec_-SW2 mCh(Gro_L2_) TetR-tetAP IncS_Ct_-3×FLAG *incDEFG* terminator after the removal of mCh(Gro) by AgeI digestion, generating the plasmid p2TK2_Spec_-SW2 GFP(nmP) TetR-tetAP IncS_Ct_-3×FLAG *incDEFG* terminator.

### Construction of p2TK2_Spec_-SW2 GFP(nmP) TetR-tetA^P^ IncS_Cm_-3×FLAG *incDEFG* terminator.

A DNA fragment (PCR A) corresponding to the *tet* repressor (TetR) and *tetA* promoter (*tetA^P^*) was amplified by PCR from p2TK2-SW2 mCh(Gro_L2_) Tet-IncV-3F plasmid (24) using primers TetRSTOP5Kpn and Tet tc0424 Rv. A DNA fragment (PCR B) corresponding to the *tc0424* (*incS_Cm_*) ORF was amplified from C. muridarum genomic DNA by PCR using primers Tet tc0424 Fw and tc0424 NotI Rv. A DNA fragment (PCR C) corresponding to TetR-tetAP IncS_Cm_ was amplified by overlapping PCR using primers TetRSTOP5Kpn and tc0424 NotI Rv and cloned into the KpnI/NotI sites of p2TK2_Spec_-SW2 mCh(Gro_L2_) MCS-3×FLAG (46), generating the plasmid p2TK2_Spec_-SW2 mCh(Gro_L2_) TetR-tetAP IncS_Cm_-3×FLAG *incDEFG* terminator. Then, mCherry was removed by AgeI digestion and GFP(nmP) was cloned into the AgeI restriction site.

### Construction of p2TK2_Spec_-SW2 mCh(Gro_L2_) TetR-tetA^P^ IncS_Ct_-HA *incDEFG* terminator.

A DNA fragment corresponding to the HA and the *incDEFG* operon terminator was amplified by PCR from p2TK2_Spec_-SW2 mCh(Gro_L2_) MCS-3×FLAG (46) using primers NotI-HA-incD term Fw and IncDTermRv and cloned into the NotI/SalI sites of p2TK2_Spec_-SW2 mCh(Gro_L2_) (46), to generate p2TK2_Spec_-SW2 mCh(Gro_L2_) MCS-HA. A DNA fragment corresponding to the *tet* repressor (TetR), *tetA* promoter (*tetA^P^*), and *incS_Ct_* ORF was amplified by PCR from plasmid p2TK2_Spec_-SW2 mCh(Gro_L2_) TetR-tetAP IncS_Ct_-3×FLAG *incDEFG* terminator using primers TetRSTOP5Kpn and IncSNotI Rv and was then cloned into the KpnI/NotI sites of p2TK2_Spec_-SW2 mCh(Gro_L2_) MCS-HA. The resulting plasmid, p2TK2_Spec_-SW2 mCh(Gro_L2_) TetR-tetAP IncS_Ct_-HA *incDEFG* terminator, will be referred as pTet-IncS_Ct_.

### Construction of pSUmC IncS_Ct_-IncS_Cm_-3×FLAG SWAP.

Three-kilobase DNA fragments downstream and upstream of *incS_Ct_* (PCR A, right arm, and PCR B, half left arm, respectively) were amplified from C. trachomatis L2 genomic DNA via PCR using primers pSUmC3Dwn0402 5 2.1 and 3Dwn0402pSumC 3 2.1 and primers pSUmC3Up0402 5 and 3Up0402TC0424 3, respectively. A DNA fragment corresponding to IncS_Cm_-3×FLAG (PCR C) was amplified by PCR from p2TK2_Spec_-SW2 GFP(nmP) TetR-tetA^P^ IncS_Cm_-3×FLAG *incDEFG* terminator using primers 3 Up0402TC0424 5 and 3xFLAG-pSUmC 3. The DNA fragment corresponding to 3-kb upstream *incS_Ct_-incS_Cm_*-3×FLAG (PCR D, complete left arm) was amplified by overlapping PCR using primers pSUmC3Up0402 5 and 3×FLAG-pSUmC 3. Gibson assembly reaction using HiFi DNA assembly master mix (New England BioLabs) and following manufacturer instructions was used to sequentially clone PCR A (right arm) into the SbfI site and PCR D (left arm) into the SalI site of pSUmC-4.0 (32) so that each arm immediately flanked the *aadA-gfp* cassette.

### Generation of the C. trachomatis SWAP strains.

C. trachomatis L2 was transformed via CaCl_2_ and isolated by plaque purifications as previously described (46). Floxed cassette allelic exchange mutagenesis via FLAEM was accomplished as described previously (34) with some modifications. Briefly, 1 μg of pSUmC IncS_Ct_-IncS_Cm_-3×FLAG SWAP plasmid was used to transform wild-type C. trachomatis L2, and transformants were selected with 500 μg/mL of spectinomycin and 50 ng/mL of aTc. Generation of the C. trachomatis SWAP strain was accomplished by first cultivating pSUmC IncS_Ct_-IncS_Cm_-3×FLAG SWAP-*aadA-gfp* transformants in the absence of aTc for multiple passages at a low multiplicity of infection (MOI; ~0.1), followed by plaque purification of Spec-resistant GFP-expressing chlamydiae. The allelic replacement was confirmed by PCR with the IncS 3-kb Ups Fwd and IncS 3-kb Dwn Rv primers (Table S1). The resulting strain was transformed with the plasmid pSU-Cre carrying ampicillin resistance (32). After 3 passages in the presence of 0.5 U penicillin G (PenG) and 50 ng/mL of aTc, the green fluorescence was lost. The excision of the *aadA-gfp* selection cassette was verified by PCR with the primers IncS 3 kb Ups Fwd and IncS 3 kb Dwn Rv (Fig. S2B) and TC0424 (3469–3489) and IncS Dwn Rv (Fig. S2B and C; Table S1) and DNA sequencing (Fig. S2D). The strain was cured of the pSU-Cre plasmid after performing 4 passages in the absence of aTc, denoted by the loss of mCherry expression (colorless inclusions). The colorless bacteria were plaque purified and amplified. The resulting strain was transformed with the p2TK2_Spec_-SW2 mCh(Gro_L2_) plasmid (46) or the complementation plasmid p2TK2_Spec_-SW2 mCh(Gro) Tet-IncS_Ct_-HA. The resulting strains were named C. trachomatis SWAP and C. trachomatis SWAP+pTet-IncS_Ct_, respectively.

### DNA transfection.

DNA transfection was performed using X-tremeGENE 9 DNA transfection reagent (Roche), according to manufacturer’s recommendations.

### Vectors for expression in mammalian cells.

STIM1-3×FLAG, CFP-STIM1, CFP-VAPA, CFP-CERT, mCh-STIM1 (1–685), mCh-STIM1 (1–535), mCh-STIM1 (1–246), mCh-STIM1 (Δ253–535), and GFP-STIM1 (234–535) plasmids were previously described (26).

### Coimmunoprecipitation.

For mass spectrometry samples, 6 × 10^6^ HEK293 cells expressing STIM1-3×FLAG seeded in 10-cm^2^ dishes were infected for 24 h with C. trachomatis (MOI of 2) or not (control). At 23 h p.i. cells were incubated with 300 μM lactacystin (Sigma) for 1 h, washed once with phosphate-buffered saline (PBS), and lysed in 2 mL of lysis buffer (20 mM Tris [pH 7.5], 150 mM NaCl, 2 mM EDTA, 1% Triton X-100, protease inhibitor cocktail EDTA-free [Roche]) for 20 min at 4°C, rotating. All subsequent steps were performed at 4°C. The lysates were centrifuged at 13,000 rpm for 10 min, and the supernatants were incubated with 20 μL of anti-FLAG M2 affinity beads (Sigma) for 2 h, rotating. The protein-bound beads were washed three times with wash buffer (20 mM Tris [pH 7.5], 150 mM NaCl, 2 mM EDTA, 1% Triton X-100), and proteins were eluted in 40 μL of elution buffer (20 mM Tris [pH 7.5], 150 mM NaCl, 2 mM EDTA, 100 μg/mL 3×FLAG peptide [Sigma]) and separated via 10% SDS-PAGE. Samples were sliced for gel digestion and analyzed at the W.M. Keck Biomedical Mass Spectrometry Laboratory of the University of Virginia. We note that the experiment described above was designed as a pilot experiment that did not include biological or technical replicates. C. trachomatis proteins were not detected in the uninfected sample. Fewer than 30 C. trachomatis proteins were detected in the infected samples. More than one peptide were detected for only four proteins, namely, DnaK (21 peptides), IncS (16 peptides), FliN (10 peptides), and RpoC (5 peptides).

For coimmunoprecipitation experiments, the protocol was slightly modified. HEK293 or HeLa cells (5 × 10^5^) were seeded in 6-well tissue culture plates and transfected (1 μg DNA/well) for 24 h prior to C. trachomatis infection (MOI of 5). Infected cells were incubated in the presence of 20 ng/mL of aTc for 4 h prior to being lysed in 250 μL of lysis buffer. A 20-μL aliquot of the precleared lysates was collected (lysate), and samples were eluted in 30 μL (IP).

### Immunoblotting.

Protein samples were separated by SDS-PAGE and transferred to nitrocellulose membranes. The membranes were stained with a Ponceau S solution, to ensure even transfer, and rinsed with distilled water (dH_2_O) before blocking in blocking buffer (5% nonfat milk in 1× PBS, 0.05% Tween) for 1 h at room temperature. Primary and horseradish peroxidase (HRP)-conjugated secondary antibodies were diluted in blocking buffer and incubated with the membranes overnight (ON) at 4°C and for 1 h at room temperature, respectively. HRP-conjugated secondary antibodies were detected with the Amersham ECL Western blotting detection reagent according to the manufacturer’s recommendations and a Bio-Rad ChemiDoc imaging system.

### Immunofluorescence, confocal microscopy, and quantification of protein associated with the inclusion.

For immunofluorescence experiments, cells seeded on coverslips were transfected for 24 h prior to C. trachomatis infection (MOI of 1) and incubated in the presence of 2 ng/mL of aTc starting 4 h postinfection. C. trachomatis *incS_Ct_* conditional mutant infection was performed in the presence of 0.5 ng/mL aTc at all times otherwise indicated. At the indicated times, samples were fixed with 4% paraformaldehyde in PBS for 30 min. All steps were performed at room temperature. Coverslips were sequentially incubated with primary and secondary antibodies diluted in 0.1% Triton X-100 in 1× PBS for 1 h. For CT147 staining cells were fixed with cold methanol (MeOH) for 10 min and then incubated ON at 4°C with anti-CT147. Coverslips were washed with 1× PBS and mounted with DABCO antifade-containing mounting medium. Imaging was performed using the Leica DMi8 microscope equipped with the Andor iXon Ultra 888BV electron-multiplying charge-coupled device (EMCCD) camera and the confocal scanner unit CSU-W1 and driven by the IQ software. Images were processed using the Imaris software (Bitplane, Belfast, United Kingdom) as follows. For quantification, 3-dimensional reconstructions of the raw signal corresponding to each marker were generated using the Imaris imaging software as previously described (24). For each marker, the volume corresponding to the sum of the pixels, above the threshold corresponding to the signal in the cytoplasm, was determined. The host protein volumes were normalized to the corresponding inclusion volume to determine the inclusion association of the respective markers in arbitrary units. At least 30 inclusions were quantified per condition. Each experiment was performed in triplicate. One representative experiment is shown. The graphs were generated using GraphPad Prism. Statistical analysis was performed using the appropriate test as indicated in the figure legends.

### Antibodies.

The following primary antibodies were used for immunofluorescence (IF) microscopy and Western blotting (WB): mouse anti-CT147 (kind gift from Guangming Zhong, UTHSCSA) (1:50 for IF), mouse monoclonal anti-FLAG (1:1,000 for IF; 1:10,000 for WB; Sigma), rabbit polyclonal anti-GFP (1:2,000 for WB; Invitrogen), rabbit polyclonal anti-mCherry (1:2,000 for WB; BioVision), rabbit polyclonal anti-HA (1:300 for IF; Sigma). The following secondary antibodies were used: Alexa Fluor 594-conjugated goat anti-mouse antibody, Alexa Fluor 514-conjugated goat anti-mouse antibody, Alexa Fluor PB-conjugated goat anti-rabbit antibody (all 1:500 for IF; Molecular Probes), peroxidase-conjugated goat anti-mouse IgG and anti-rabbit IgG (both 1:10,000 for Western blotting; Jackson ImmunoResearch).

### Infectious progeny production.

To determine the infectious progeny production, infected HeLa cells in 96-well plates were lysed 48 h postinfection in 100 μL sterile water, and 5-fold dilutions of the lysates were used to infect fresh HeLa cell monolayers. Infected cells were stained with Hoechst stain. The number of infected cells was determined by automated imaging using an ImageXpress automated system. Quantification of inclusion-forming units (IFUs) per milliliter was determined using the MetaXpress software. The fold growth was determined as the ratio between the IFUs per milliliter at 48 h and those at 0 h p.i.

### Lysis assay.

Confluent monolayers of HeLa cells or wild-type or STIM1/2 DKO MEFs plated in 96 wells were infected with the indicated C. trachomatis strains at an MOI of 0.005. To limit overgrowth of the MEF cells over the 5-day period of the assay, lysis assay in MEF cells was carried out in the presence of 1 μg/mL cycloheximide. Cycloheximide was not used to conduct the lysis assay in HeLa cells. At the indicated time points, the number of intact and lysed inclusions was manually quantified live with a fluorescence microscope, as described in the work of Bishop and Derré (43). The percentage of lysed inclusions was determined as the number of lysed inclusions/total of inclusions. One hundred to 150 inclusions were quantified per well, with 4 biological replicates per experiment.

### Statistics.

Each experiment was performed in triplicate. The sample size is indicated in the figure legends. The average ± standard error of the mean (SEM) from one representative experiment is shown. The graphs were generated using GraphPad Prism. The appropriate statistical tests were used and are indicated in the figure legends.
