# Supplementary figures and images for: Homologues of the Chlamydia trachomatis and Chlamydia muridarum Inclusion Membrane Protein IncS Are Interchangeable for Early Development but Not for Inclusion Stability in the Late Developmental Cycle
Source: mSphere. 2023 Feb 28;8(2):e00003-23. doi: 10.1128/msphere.00003-23 (PMC10117133; doi:10.1128/msphere.00003-23)

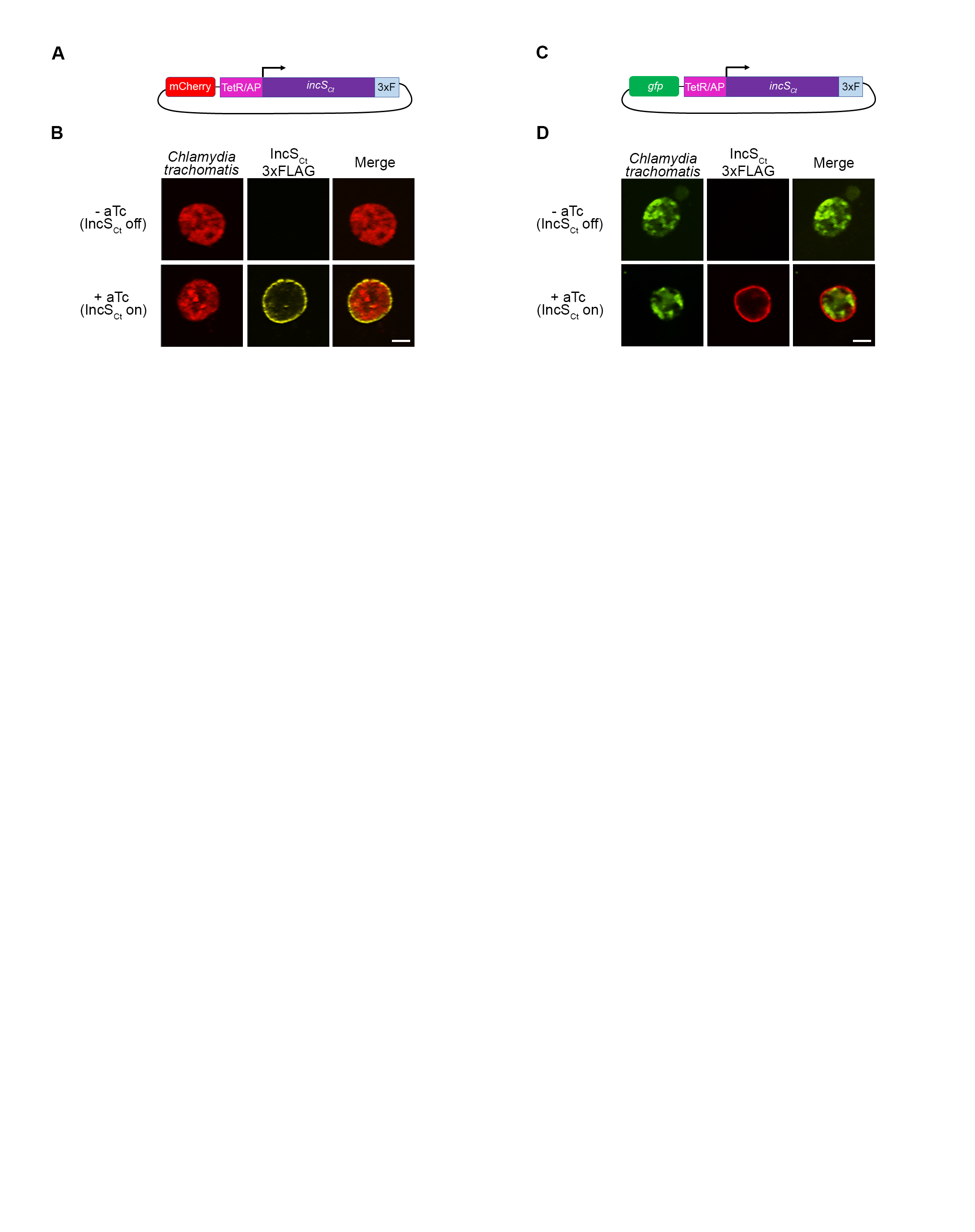

Supplement: FIG S1 [file msphere.00003-23-s0001.tif]

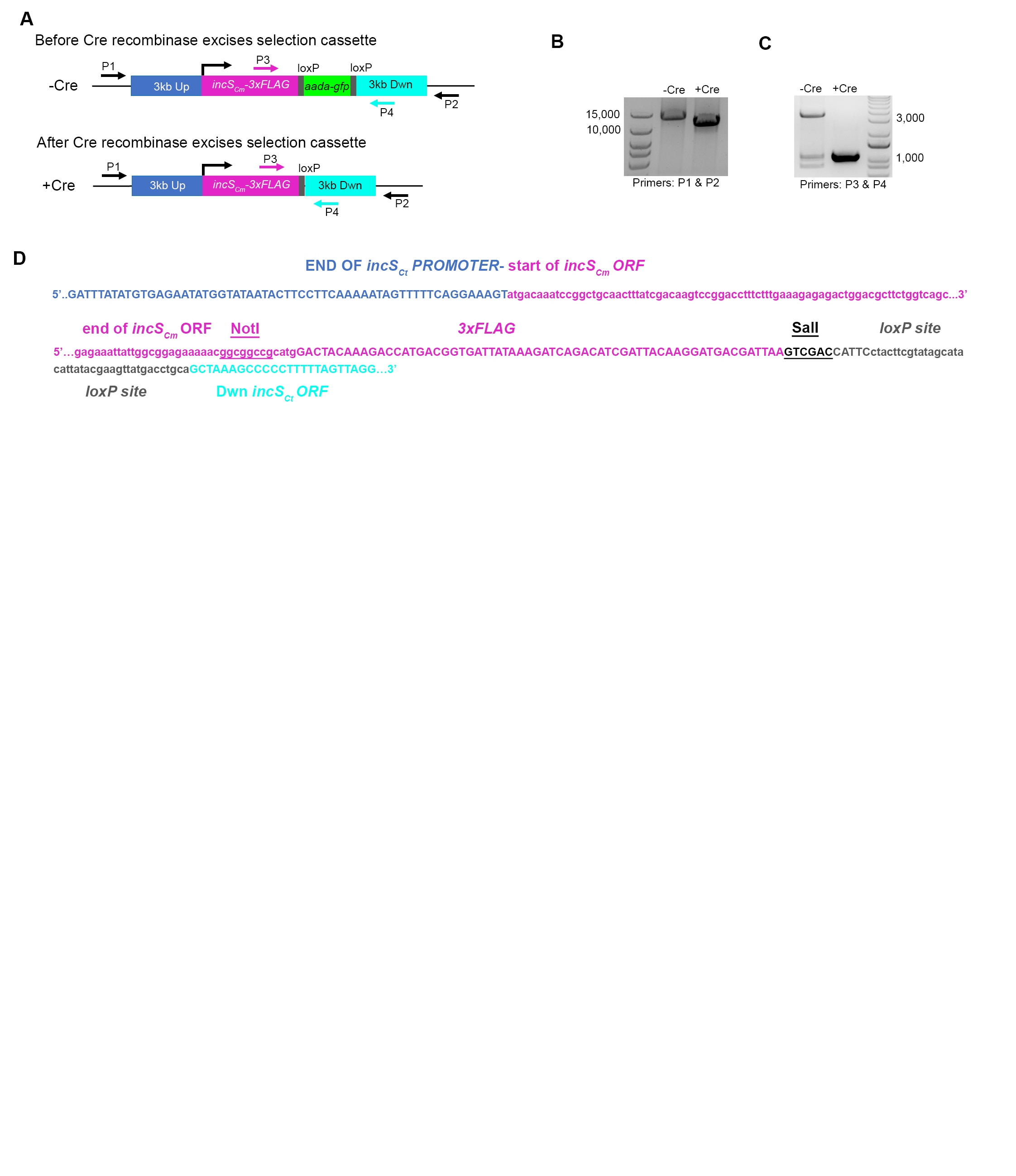

Supplement: FIG S2 [file msphere.00003-23-s0002.tif]

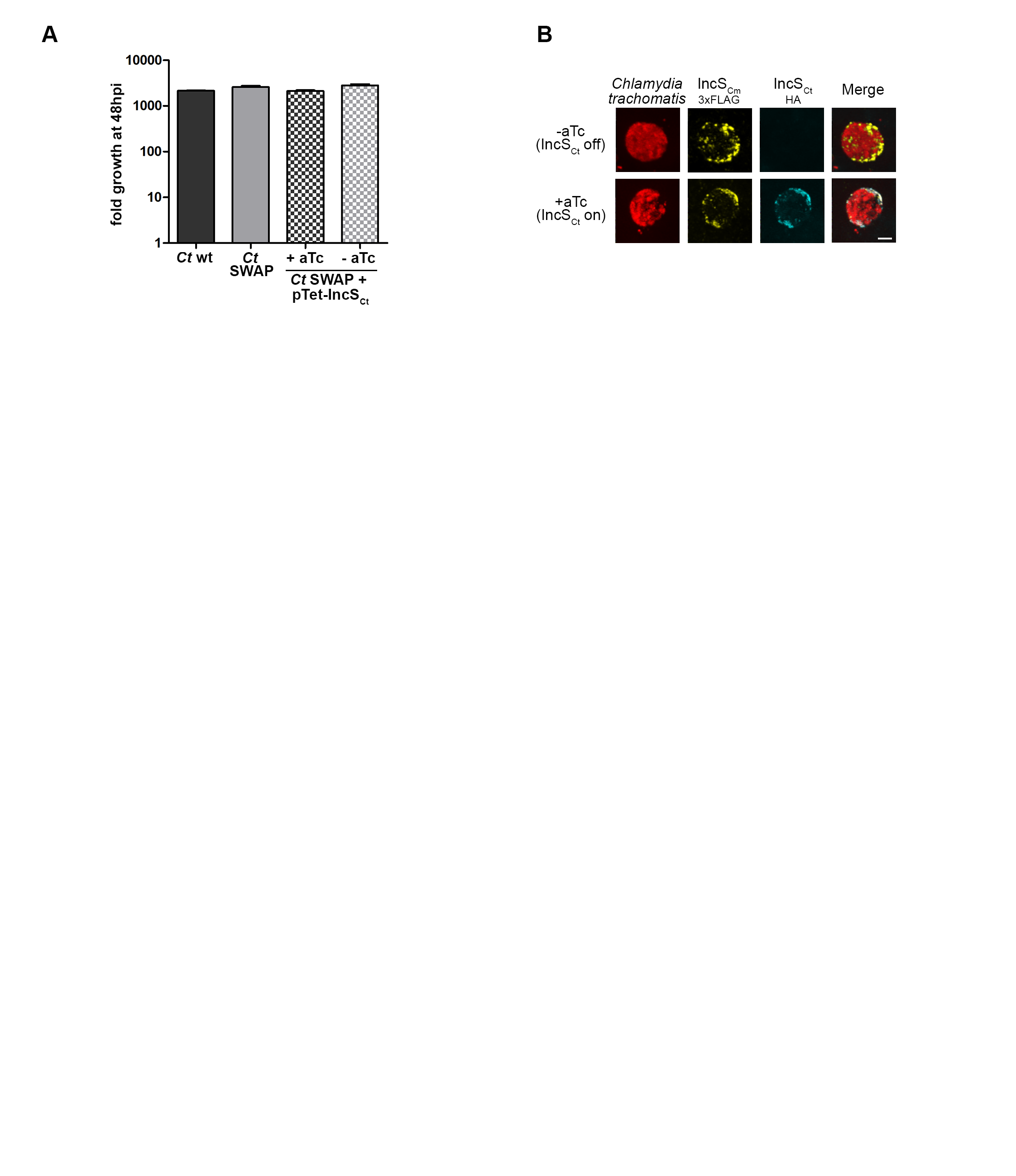

Supplement: FIG S3 [file msphere.00003-23-s0003.tif]

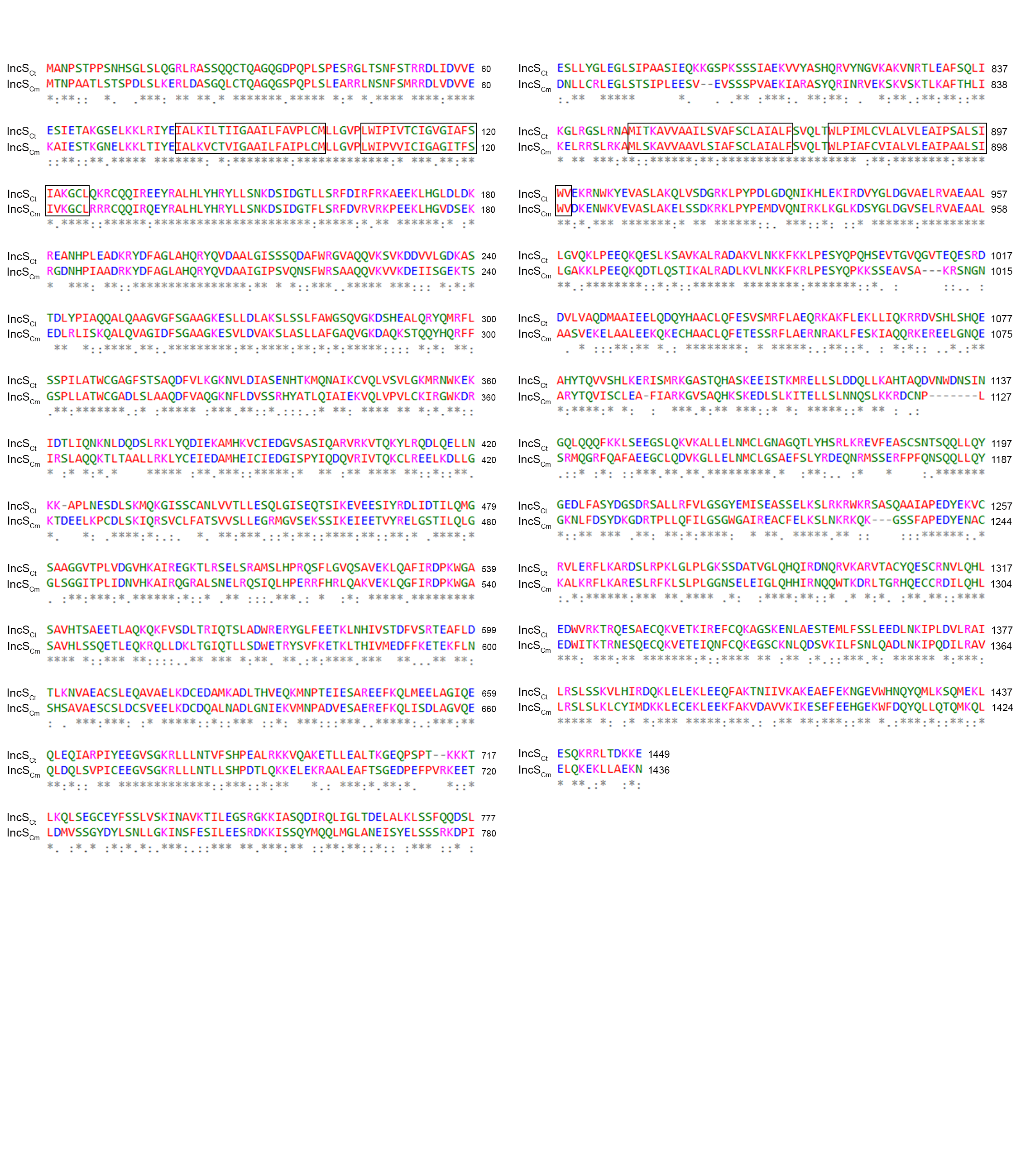

Supplement: FIG S5 [file msphere.00003-23-s0005.tif]

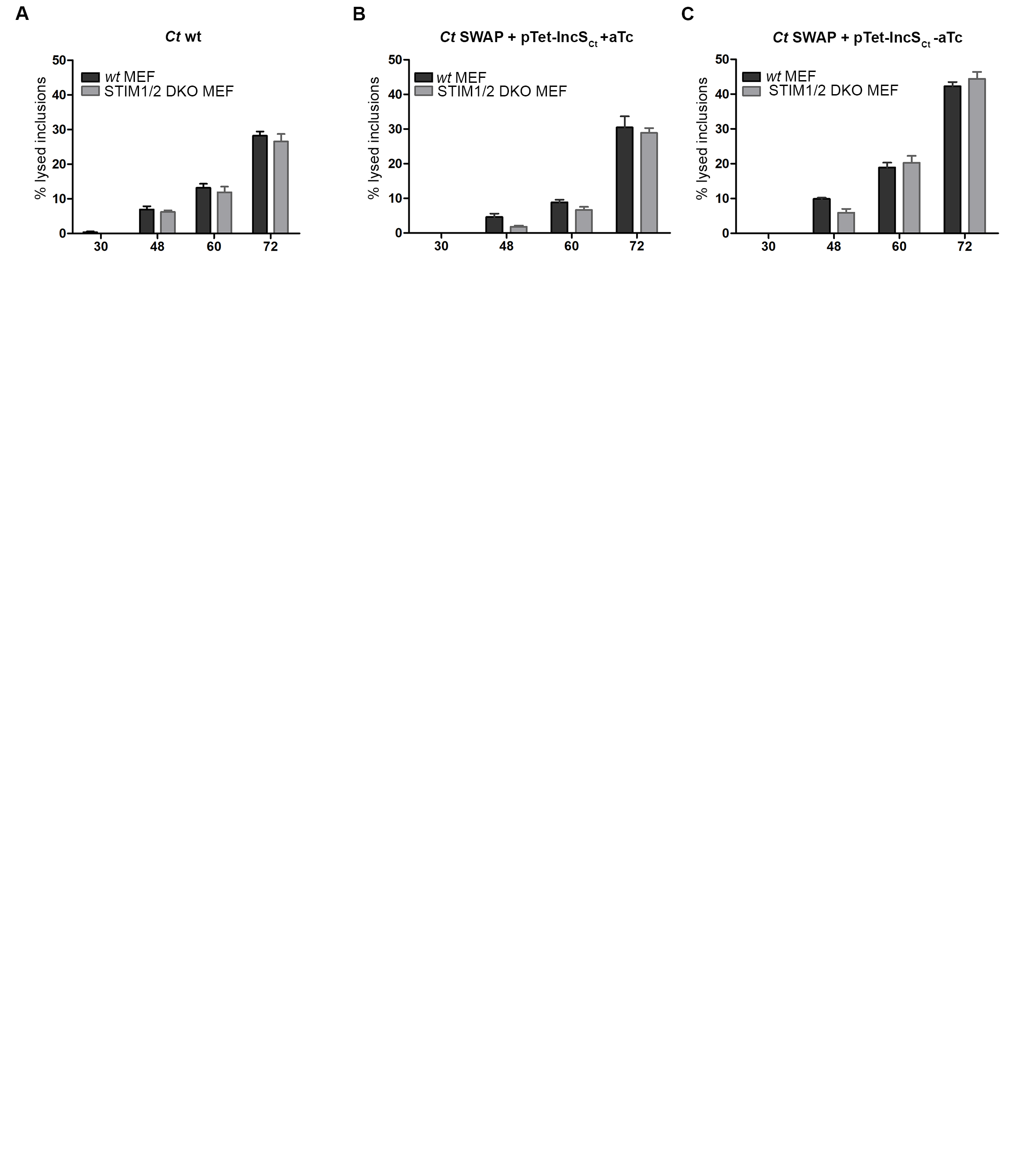

Supplement: FIG S4 [file msphere.00003-23-s0004.tif]
