# Supplementary material for: Homologues of the Chlamydia trachomatis and Chlamydia muridarum Inclusion Membrane Protein IncS Are Interchangeable for Early Development but Not for Inclusion Stability in the Late Developmental Cycle
Source: mSphere. 2023 Feb 28;8(2):e00003-23. doi: 10.1128/msphere.00003-23 (PMC10117133; doi:10.1128/msphere.00003-23)
Supplement: TABLE S1 [file msphere.00003-23-s0006.docx]

| **CLONING** |  |  |  |
| --- | --- | --- | --- |
| **p2TK2_Spec_-SW2 mCh(Gro_L2_) TetR-tetA^P^ IncS_Ct_-3xFLAG *incDEFG* terminator** | | |  |
| Constructed by overlapping PCR | | |  |
| **PCR:** | **Primer Names:** | **Primer Sequences:** | **Template:** |
| PCR A | TetRSTOP5Kpn | GGTGGTACCTTAAGACCCACTTTCACATTTAAG | p2TK2-SW2 mCh(Gro_L2_) Tet-IncV-3F plasmid |
|  | TetAP-IncS Rv | ATTGGATGGGGGTGTAGACGGATTCGCCAT  ttcacttttctctatcactgatagggagtg |  |
| PCR B | TetAP-IncS Fw | cactccctatcagtgatagagaaaagtgaaATGGCGAATC  CGTCTACACCCCCATCCAAT | *CtL2* genomic DNA |
|  | 0402 FLAG Rv | CATGGTCTTTGTAGTCcatTTCTTTCTTATCTGTCAGTC |  |
| PCR C | 0402 FLAG Fw | GACTGACAGATAAGAAAGAAatgGACTACAAAGACCATG | p2TK2-SW2 mCh(Gro_L2_) Tet-IncV-3F plasmid |
|  | IncDTerm 3 Not | GCGGGCGGCCGCgtcttaggagctttttgcaatgc |  |
| PCR D | TetRSTOP5Kpn | GGTGGTACCTTAAGACCCACTTTCACATTTAAG | PCR A + PCR B + PCR C |
|  | IncDTerm 3 Not | GCGGGCGGCCGCgtcttaggagctttttgcaatgc |  |
|  |  |  |  |
| \| **p2TK2_Spec_-SW2 GFP(nmP) TetR-tetA^P^ IncS_Ct_-3xFLAG *incDEFG* terminator** \| \| --- \| \| Constructed by swapping mCherry for GFP into the AgeI restriction site \| | | | |
| **PCR:** | **Primer Names:** | **Primer Sequences:** | **Template:** |
| PCR E | AgeI nmP Prom Fw | accACCGGTGATGCCCGACGGTCTTTATAGCG | pGFP::SW2 |
|  | RSGFP TAA AgeI Rv | accACCGGTTTAAGATCTGAGTCCGGACTTGTATAG |  |
|  |  |  |  |
| **p2TK2_Spec_-SW2 GFP(nmP) TetR-tetA^P^ IncS_Cm_-3xFLAG *incDEFG* terminator** | | |  |
| Constructed by overlapping PCR | | |  |
| **PCR:** | **Primer Names:** | **Primer Sequences:** | **Template:** |
| PCR A | TetRSTOP5Kpn | GGTGGTACCTTAAGACCCACTTTCACATTTAAG | p2TK2-SW2 mCh(Gro) Tet-IncV-3F plasmid |
|  | Tet tc0424 Rv | GTTGCAGCCGGATTTGTCATttcacttttctctatcactg |  |
| PCR B | Tet tc0424 Fw | cagtgatagagaaaagtgaaATGACAAATCCGGCTGCAAC | *C muridarum* genomic DNA |
|  | tc0424 NotI Rv | gcg**GCGGCCGC**cGTTTTTCTCCGCCAATAATTTC |  |
| PCR C | TetRSTOP5Kpn | GGTGGTACCTTAAGACCCACTTTCACATTTAAG | PCR A + PCR B |
|  | tc0424 NotI Rv | gcg**GCGGCCGC**cGTTTTTCTCCGCCAATAATTTC |  |
|  |  |  |  |
| **pSUmC IncS_Ct_-IncS_Cm_-3xFLAG SWAP** | | |  |
| Constructed by Gibson assembly | | |  |
| **PCR:** | **Primer Names:** | **Primer Sequences:** | **Template:** |
| PCR A (right arm) | pSUmC3Dwn0402 5 2.1 | CGTATAGCATACATTATACGAAGTTATGacctgca  GCTAAAGCCCCCTTTTTAGTTAG | *CtL2* genomic DNA |
|  | 3Dwn0402pSumC 3 2.1 | ctttGATCTTTCTACGGGGTCTGACGCcctgca  TCGCATACTTTAGACACGGTGAG |  |
| PCR B | pSUmC3Up0402 5 | cgtcaCTGCAGGTACCGGGCATGCTTTTGTATTAGTAGAG | *CtL2* genomic DNA |
|  | 3Up0402TC0424 3 | gttgcagccggatttgtcatACTTTCCTGAAAAACTATTTTTGAAGG |  |
| PCR C | 3 Up0402 TC0424 5 | CCTTCAAAAATAGTTTTTCAGGAAAGTatgacaaatccggctgcaac | p2TK2_Spec_-SW2 GFP(nmP) TetR-tetAP IncS_Cm_-3xFLAG incDEFG terminator |
|  | 3xFlag-pSUmC 3 | gctatacgaagtagGAATGGTCGACTTAATCGTC  ATCCTTGTAATCG |  |
| PCR D (left arm) | pSUmC3Up0402 5 | cgtcaCTGCAGGTACCGGGCATGCTTTTGTATTAGTAGAG | PCR B + PCR C |
|  | 3xFlag-pSUmC 3 | gctatacgaagtagGAATGGTCGACTTAATCGTCATC  CTTGTAATCG |  |
|  |  |  |  |
| **p2TK2_Spec_-SW2 mCh(Gro_L2_) MCS-HA *incDEFG* terminator** | | |  |
| **PCR:** | **Primer Names:** | **Primer Sequences:** |  |
| PCRA | NotI-HA-incD term Fwd | gcgGCGGCCGCatgtacccatacgatgttccagattacgctTAA  ggatgacatgtgattcgcgtag |  |
|  | IncD Term Rv | **GTCGTCGAC**gtcttaggagctttttgcaatgc |  |
|  |  |  |  |
| **p2TK2_Spec_-SW2 mCh(Gro_L2_) TetR-tetA^P^ IncS_Ct_-HA *incDEFG* terminator** | | |  |
| **PCR:** | **Primer Names:** | **Primer Sequences:** |  |
| PCRA | TetRSTOP5Kpn | GGTGGTACCTTAAGACCCACTTTCACATTTAAG |  |
|  | IncS NotI Rv | gcgGCGGCCGCcTTCTTTCTTATCTGTCAGTC |  |

| **SEQUENCING** |  |
| --- | --- |
| **p2TK2_Spec_-SW2 GFP(nmP) TetR-tetA^P^ IncS_Ct_-3xFLAG *incDEFG* terminator** | |
| **Primer Names:** | **Primer Sequences:** |
| CTL0402 (M78) KpnI Rv | ggtGGTACCTTACTCATATATTCGAAGTTTTTTAAG |
| pMyc-EcoR1-0402 (M127) Fw | gaaGAATTCggCAGAAAAGATGTCAGCAGATTC |
| pMyc-EcoRI-0402 (M400) Fw | gaaGAATTCgg CAG GCT CGG GTG CGT AAG GTC |
| CTL0402 (M899) KpnI Rv | ggtGGTACCTTACACCCAAATAGATAAAGCAGAAGG |
| pMyc-EcoR1-0402 (M846) Fw | gaaGAATTCggAATGCGATGATCACTAAAGC |
| pMyc-EcoRI-0402 (M900) Fw | gaaGAATTCggGAGAAAAGAAACTGGAAATATG |
| 0402 FLAG Rv | CATGGTCTTTGTAGTCcatTTCTTTCTTATCTGTCAGTC |
| IncDTerm 3 Not | GCGGGCGGCCGCgtcttaggagctttttgcaatgc |
| 2TK2 SW2 Spec Rev | GTGGGTGTTTGTACTAGAGG |
| Gro mCh STOP 5 | GGACGAGCTGTACAAGTAGttcctctaatgggaacaaatag |
|  |  |
| **p2TK2_Spec_-SW2 GFP(nmP) TetR-tetA^P^ IncS_Cm_-3xFLAG *incDEFG* terminator** | |
| **Primer Names:** | **Primer Sequences:** |
| TetRTetAP_MEC Fw | CTAGACATcattaattcctaatttttgttg |
| TC0424 (604-629) | CGTTATCAGGTAGATGCCGCTATTGG |
| TC0424 (1310-1333) | GCGTCTGTTTGTTTGCAACTTCCG |
| TC0424 (2032-2053) | CGTCTCTTATTGAATACACTGC |
| TC0424 (2748-2763) | GTCTTCAGACAAAAGGAAGC |
| TC0424 (3469-3489) | GGTTCCGCCGAATTTTCTCTG |
| IncD Term Rv | GTCGTCGACgtcttaggagctttttgcaatgc |
|  |  |
| **pSUmC-IncS-TC0424-3xFLAG SWAP** | |
| **Primer Names:** | **Primer Sequences:** |
| pSUmC4.0pgp6SeqFwd | CTTCGCATCATGTGTTCCGGAG |
| 3kbUpSeqFwd1 | GAGCTATCCTTCTAGTATACTGGG |
| 3kbUpSeqFwd2 | GAGACTCCCTCAAGAGGCTC |
| 3kbUpSeqFwd3 | GGTGTCGGTGCGTTGTATCAATC |
| 3kbUpSeqFwd4 | GGAACGAGAGCAAATTATCAAG |
| TC0424 (604-629) | CGTTATCAGGTAGATGCCGCTATTGG |
| TC0424 (1310-1333) | GCGTCTGTTTGTTTGCAACTTCCG |
| TC0424 (2032-2053) | CGTCTCTTATTGAATACACTGC |
| TC0424 (2748-2763) | GTCTTCAGACAAAAGGAAGC |
| TC0424 (3469-3489) | GGTTCCGCCGAATTTTCTCTG |
| pSUmC4.0aadASeqRev | ggcttatgtccactgggttcg |
| SUmC-bla-gfp Fw | ccattatcaacaaaatactccaattggcgatgg |
| 3kbDwn2.1SeqFwd1 | CATCAGGGCTGGAGTATACATG |
| 3kbDwn2.1SeqFwd2 | GGGGCAACGAGAGGCTGATG |
| 3kbDwn2.1SeqFwd3 | CTCGACACAGCTTGAGGGGAAG |
| pSUmC4.0ORISeqRev | caaaccaccgctggtagcggtg |
|  |  |
| ***Ct* SWAP strain** | |
| **Primer Names:** | **Primer Sequences:** |
| IncS 3kb Ups Fwd | GGGATGGGTTTATCTTAAGTTG |
| IncS 3kB Dwn Rv | GGTTTTTGCTGGCCATGAATGAACC |
| TC0424 (3469-3489) | GGTTCCGCCGAATTTTCTCTG |
| IncS Dwn Rv | CGCACACATTCAAGTTGCATGAGC |
|  |  |
